# Astataricusones A–D and Astataricusol A, Five New Anti-HBV Shionane-Type Triterpenes from *Aster tataricus* L. f.

**DOI:** 10.3390/molecules181214585

**Published:** 2013-11-25

**Authors:** Wen-Bing Zhou, Guang-Zhi Zeng, Hui-Min Xu, Wen-Jun He, Ning-Hua Tan

**Affiliations:** 1 State Key Laboratory of Phytochemistry and Plant Resources in West China, Kunming Institute of Botany, Chinese Academy of Sciences, Heilongtan, Kunming 650201, China; E-Mails: yaowufx2001@163.com (W.-B.Z.); gzh_zeng@mail.kib.ac.cn (G.-Z.Z.); cpuagua@126.com (H.-M.X.); hewenjun@mail.kib.ac.cn (W.-J.H.); 2 University of Chinese Academy of Sciences, Beijing 100049, China

**Keywords:** *Aster tataricus*, shionane-type triterpenes, astataricusones A–D, astataricusol A, anti-HBV

## Abstract

Five new shionane-type triterpenes, astataricusones A–D (compounds **1**–**4**) and astataricusol A (**5**), together with one known shionane-type triterpene **6** were obtained from the roots and rhizomes of *Aster tataricus* L. f. Their structures were elucidated on the basis of spectroscopic data, mainly NMR and MS data. The absolute configurations of **1** and **4** was determined by single crystal X-ray diffraction and CD analysis. Compound **2** showed inhibitory activity on HBsAg secretion with an IC_50_ value of 23.5 μM, while **2** and **6** showed inhibitory activities on HBeAg secretion with IC_50_ values of 18.6 and 40.5 μM, and cytotoxicity on HepG 2.2.15 cells with CC_50_ values of 172.4 and 137.7 μM, respectively. Compounds **2** and **6** also exhibited inhibitory activities on HBV DNA replication with IC_50_ values of 2.7 and 30.7 μM, respectively.

## 1. Introduction

**Figure 1:**
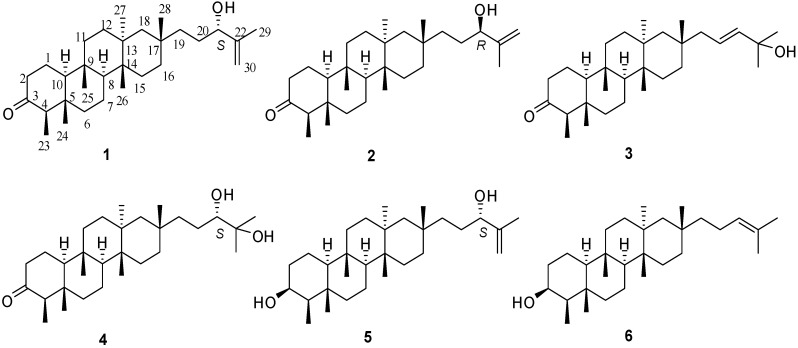
Structures of compounds **1**–**6**.

Shionane-type triterpenoids possessing a unique all six-membered tetracyclic skeleton and 3-oxo-4-monomethyl moieties have been isolated only from Compositae plants [1]. The first studies on these compounds were done in the 1960s by Ourisson and Takahashi [2,3]. The stereostructures of rings A–D were determined by total synthesis in the 1970s by Ireland [4,5], but only six ones were reported from natural resources until now [6]. It has been reported that shionane-type triterpenes are the main constituents of *Aster tataricus* L. f. and have been shown to possess antitussive and expectorant activities [1,7,8]. In 2010 three new anti-HBV shionane-type triterpenes have been reported from *A. tataricus* by us [9]. As a continuation of our work on *A. tataricus* [9,10,11,12,13], we conducted further phytochemical studies on its roots and rhizomes. This has now led to the isolation of other five new shionane-type triterpenes, astataricusones A–D **1**–**4** and astataricusol A (**5**), and a known one, epishionol (**6**) (Figure 1). Herein we describe their isolation and structural elucidation, as well as their anti-HBV activity.

## 2. Results and Discussion

The EtOAc part of the *A. tataricus* MeOH extract was subjected to silica gel, RP−18, and Sephadex LH-20 column chromatographies and semipreparative HPLC to yield five new shionane-type triterpenoids, named astataricusones A–D (compounds **1**–**4**) and astataricusol A (**5**), and the known compound epishionol (**6**) [14,15].

Compound **1** was obtained as colorless needles. It was determined to have six degrees of unsaturation corresponding to its molecular formula C_30_H_50_O_2_, as established by positive HREIMS ([M]^+^ 442.3808, calcd 442.3811) and NMR spectroscopies (Table 1 and Table 2). The IR spectrum of **1** showed absorption bands for OH (3,498 cm^−1^) and C=O (1,697 cm^−1^) functions.

**Table 1:** ^13^C-NMR spectroscopic data for **1**–**5** (*δ* in ppm, CDCl_3_, 100 MHz).

Position	1	2	3	4	5
1	22.3, CH_2_	22.2, CH_2_	22.3, CH_2_	22.3, CH_2_	15.8, CH_2_
2	41.4, CH_2_	41.4, CH_2_	41.4, CH_2_	41.4, CH_2_	35.2, CH_2_
3	213.3, C	213.1, C	213.2, C	213.3, C	72.7, CH
4	58.1, CH	58.1, CH	58.1, CH	58.2, CH	49.1, CH
5	42.1, C	42.1, C	42.1, C	42.1, C	37.8, C
6	41.0, CH_2_	41.1, CH_2_	41.0, CH_2_	41.1, CH_2_	41.6, CH_2_
7	17.8, CH_2_	17.8, CH_2_	17.9, CH_2_	17.9, CH_2_	17.2, CH_2_
8	49.8, CH	49.8, CH	49.8, CH	49.9, CH	49.9, CH
9	38.4, C	38.4, C	38.4, C	38.4, C	38.1, C
10	59.5, CH	59.6, CH	59.5, CH	59.6, CH	61.5, CH
11	35.2, CH_2_	35.2, CH_2_	35.2, CH_2_	35.2, CH_2_	35.1, CH_2_
12	32.2, CH_2_	32.2, CH_2_	32.1, CH_2_	32.2, CH_2_	32.4, CH_2_
13	36.8, C	36.8, C	36.9, C	36.9, C	36.8, C
14	38.5, C	38.5, C	38.4, C	38.6, C	38.6, C
15	29.1, CH_2_	29.1, CH_2_	29.2, CH_2_	29.1, CH_2_	29.1, CH_2_
16	34.7, CH_2_	34.5, CH_2_	33.8, CH_2_	34.7, CH_2_	34.7, CH_2_
17	31.3, C	31.4, C	32.1, C	31.6, C	31.4, C
18	44.4, CH_2_	44.4, CH_2_	45.3, CH_2_	44.5, CH_2_	44.5, CH_2_
19	38.9, CH_2_	38.8, CH_2_	45.8, CH_2_	26.5, CH_2_	38.9, CH_2_
20	29.7, CH_2_	25.6, CH_2_	124.2, CH	40.5, CH_2_	29.8, CH_2_
21	76.9, CH	90.5, CH	140.7, CH	79.5, CH	77.0, CH
22	147.4, C	143.5, C	70.8, C	73.2, C	147.4, C
23	6.8, CH_3_	6.8, CH_3_	6.8, CH_3_	6.8, CH_3_	11.6, CH_3_
24	14.6, CH_3_	14.6, CH_3_	14.6, CH_3_	14.6, CH_3_	16.4, CH_3_
25	19.6, CH_3_	19.5, CH_3_	19.6, CH_3_	19.6, CH_3_	20.0, CH_3_
26	15.1, CH_3_	15.1, CH_3_	15.1, CH_3_	15.2, CH_3_	15.0, CH_3_
27	20.5, CH_3_	20.5, CH_3_	21.0, CH_3_	20.6, CH_3_	20.6, CH_3_
28	33.0, CH_3_	32.7, CH_3_	32.9, CH_3_	32.9, CH_3_	33.0, CH_3_
29	17.3, CH_3_	17.0, CH_3_	29.9, CH_3_	23.2, CH_3_	17.3, CH_3_
30	111.2, CH_2_	114.6, CH_2_	29.9, CH_3_	26.5, CH_3_	111.2, CH_2_

**Figure 2:**
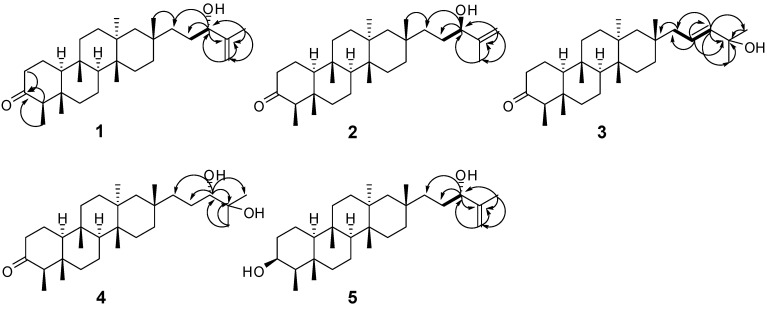
Key ^1^H−^1^H COSY (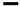) and HMBC (H
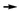
C) correlations of **1**–**5**.

The ^13^C-NMR spectrum of **1** displayed 30 carbon signals, corresponding to seven methyls (*δ*_C_ 6.8, 14.6, 15.1, 17.3, 19.6, 20.5, 33.0), twelve methylenes (including one olefinic carbon (*δ*_C_ 111.2)), four methines (including one oxygenated CH (*δ*_C_ 76.9) and seven quaternary carbons (including one keto carbonyl (*δ*_C_ 213.3) and one olefinic carbon (*δ*_C_ 147.4)) (Table 1). The ^1^H-NMR spectrum showed one secondary methyl (*δ*_H_ 0.84, 3H, d, *J* = 6.6 Hz) and six tertiary methyls (*δ*_H_ 0.68, 0.89, 1.08, 1.69 (each 3H, s), 0.86 (6H, s)) (Table 2). The above facts suggested that **1** was a tetracyclic triterpenoid with a keto carbonyl group and one terminal double bond. Comparison of the ^1^H and ^13^C-NMR data of **1** with those of shionone [16] indicated that **1** was similar to shionone in rings A–D, suggesting that the keto carbonyl group is attached to C-3, which was supported by the HMBC correlations (Figure 2): H-23 (*δ*_H_ 0.84, 3H, d, *J* = 6.6 Hz)/C-3 (*δ*_C_ 213.3), H-2 (*δ*_H_ 2.36, 1H and 2.27, 1H, m)/C-3, H-4 (*δ*_H_ 2.21, 1H, q, *J* = 6.6 Hz)/C-3. ^1^H-NMR signals of **1** at *δ*_H_ 1.20 (1H, H−19), 1.57 (1H, H-19), 1.44 (1H, H-20), 1.57 (1H, H-20), 3.97 (1H, t, *J* = 6.1 Hz, H-21), 1.69 (3H, s, H-29), 4.81 (1H, s, H-30), 4.90 (1H, s, H-30) and ^13^C-NMR signals at *δ*_C_ 38.9 (C−19), 29.7 (C-20), 76.9 (C-21), 147.4 (C-22), 17.3 (C-29), 111.2 (C-30) revealed that **1** possesses the side chain of –CH_2_-CH_2_-CH(OH)-C(=CH_2_)CH_3_, which was confirmed by the HMBC correlations between H-21 and C−19, C-20, C-22, C-29, C-30, and H-29 and C-21, C-22, C-30, and also by the ^1^H−^1^H COSY correlation between H-20 and H-21 (Figure 2). More importantly, the HMBC correlation between H-28 and C-19 indicated that C-19 was attached to C-17 (*δ*_C_ 31.3). Therefore, the planar structure of **1** was established as shown in Figure 1.

**Table 2:** ^1^H-NMR spectroscopic data for **1**–**5** (*δ* in ppm, *J* in Hz, CDCl_3_, 400 MHz).

Position	1	2	3	4	5
1a	1.95, m	1.98, m	1.98, overlap	1.98, m	1.58, overlap
1b	1.67, overlap	1.69, overlap	1.69, overlap	1.69, overlap	1.46, overlap
2a	2.36, m	2.39, m	2.38, m	2.39, m	1.90, m
2b	2.27, m	2.32, m	2.30, overlap	2.30, m	−
3	−	−	−	−	3.73, m
4	2.21, q (6.6)	2.25, q (6.6)	2.25, q (6.4)	2.25, q (6.7)	1.25, overlap
6a	1.69, overlap	1.74, overlap	1.72, overlap	1.72, overlap	1.72, overlap
6b	1.20, overlap	1.25, overlap	1.26, overlap	1.25, overlap	0.92, overlap
7a	1.47, overlap	1.47, m	1.49, m	1.50, overlap	−
7b	1.30, overlap	1.32, overlap	1.32, overlap	1.32, overlap	1.37, overlap
8	1.30, overlap	1.32, overlap	1.32, overlap	1.32, overlap	1.25, overlap
10	1.54, overlap	1.60, overlap	1.60, overlap	1.60, overlap	0.94, overlap
11a	1.51, overlap	1.53, overlap	1.53, overlap	1.50, overlap	1.58, overlap
11b	1.39, m	1.42, overlap	1.40, overlap	1.41, overlap	1.37, overlap
12a	1.54, overlap	1.53, overlap	1.53, overlap	1.60, overlap	1.56, overlap
12b	0.89, overlap	0.90, overlap	0.91, overlap	0.91, overlap	0.87, overlap
15	1.27, m	1.25, overlap	1.32, overlap	1.32, overlap	1.25, overlap
16a	1.61, overlap	1.60, overlap	1.60, overlap	1.66, overlap	1.61, overlap
16b	1.35, m	1.32, overlap	1.40, overlap	1.41, overlap	1.37, overlap
18a	1.17, overlap	1.21, d (14.4)	1.21, d (14.7)	1.25, overlap	1.19, overlap
18b	1.08, overlap	1.08, d (14.4)	1.09, d (14.7)	1.07, overlap	1.08, overlap
19a	1.57, overlap	1.78, m	2.33, overlap	1.54, overlap	1.59, overlap
19b	1.20, overlap	1.11, overlap	1.98, overlap	1.23, overlap	1.19, overlap
20a	1.57, overlap	1.60, overlap	5.66, m	1.66, overlap	1.61, overlap
20b	1.44, overlap	1.39, overlap	−	1.54, overlap	1.46, overlap
21	3.97, t (6.1)	4.26, t (6.8)	5.58, d (15.7)	3.28, d (9.7)	3.99, t (6.2)
23	0.84, d (6.6)	0.86, d (6.6)	0.87, d (6.4)	0.87, d (6.7)	0.93, d (7.3)
24	0.68, s	0.71, s	0.71, s	0.71, s	0.94, s
25	0.89, s	0.91, s	0.91, s	0.92, s	0.90, s
26	0.86, overlap	0.88, s	0.88, overlap	0.88, s	0.87, s
27	1.08, s	1.11, s	1.13, s	1.11, s	1.08, s
28	0.86, overlap	0.88, s	0.88, overlap	0.89, s	0.87, s
29	1.69, s	1.74, s	1.32, overlap	1.22, s	1.72, s
30a	4.90, s	5.05, s	1.32, overlap	1.17, s	4.92, s
30b	4.81, s	5.02, s	−	−	4.83, s

**Figure 3:**
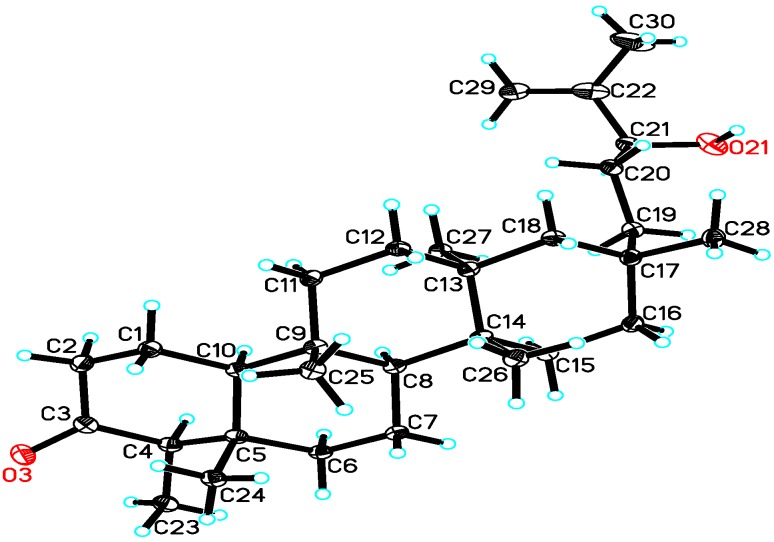
X-ray crystallographic structure of **1**.

The negative Cotton effect (−9.5) at 290 nm indicated the C-4 absolute configuration of **1** was *R* by the octant rule. In the ROESY spectrum, H-23 showed correlations to H-24, H-24 to H-25, H-26 to H-28, H-27 to H-19, which indicated H-23, H-24 and H-25, H-26 and H-28, H-27 and H-19 in the same orientation. To confirm the structure and determine its absolute configuration, **1** was crystallized from MeOH to afford a crystal of the orthorhombic space group *P*2_1_2_1_2_1_, which was analyzed by X-ray crystallography. The final refinement on the CuKα data resulted in a Flack parameter of 0.1 (3), allowing unambiguous assignment of the absolute configuration (Figure 3, CCDC 926578) [17]. These results indicated that H-23, H-24, H-25, H-26 and H-28 were *β*-oriented, and H-8, H-10, H-19 and H-27 were *α*-oriented. Therefore the absolute configuration and structure of **1** were finally assigned, *i.e.*, (4*R*,5*S*,8*S*,9*S*,10*S*,13*S*,14*R*,17*S*,21*S*)-21-hydroxy-shion-22(30)-en-3-one, and the compound was named astataricusone A.

Compound **2** was obtained as white powder, and had the same planar structure with **1**, based on the interpretation of 1D and 2D NMR data. Comparing the NMR data (Table 1 and Table 2) with those of **1** revealed that the stereostructure of C-21 was different, which was supported by the signals of C-21 at *δ*_C_ 76.9 and H-21 at *δ*_H_ 3.97 (1H, t, *J* = 6.1 Hz) in **1** and was replaced by those of C-21 at *δ*_C_ 90.5 and H-21 at *δ*_H_ 4.26 (1H, t, *J* = 6.8 Hz) in **2**. It allowed the assignment of the absolute configuration at C-21 as (*R*) in **2**. Thus, the structure of **2** was established as (4*R*,5*S*,8*S*,9*S*,10*S*,13*S*,14*R*,17*S*,21*R*)-21-hydroxy-shion-22(30)-en-3-one, and it was named astataricusone B.

Compound **3** was obtained as a white powder. Its molecular formula was determined as C_30_H_5__0_O_2_ by HREIMS at *m/z* 442.3824 [M]^+^ (calcd for 442.3811). Comparing the NMR data (Table 1 and Table 2) with those of 22-methoxy-shion-20-en-3-one [9] revealed the following differences: the presence of a hydroxyl group at C-22 was supported by the HMBC correlations between H-20 (*δ*_H_ 5.66, 1H, m), H-21 (*δ*_H_ 5.58, 1H, d, *J* = 15.7 Hz), H-29, 30 (*δ*_H_ 1.32, 6H) and C-22 in **3**, but a methoxy group at C-22 in 22-methoxy-shion-20-en-3-one. The configuration of **3** was established to be identical to **1** based on the similar ROESY correlations and CD data. The *J*_H-__20_/_H-2__1_ value (15.7 Hz) was indicative of 20*E* geometry. Hence, the structure of **3** was assigned as (4*R*,5*S*,8*S*,9*S*,10*S*,13*S*,14*R*,17*S*,20*E*)-22-hydroxy-shion-20-en-3-one, and the compound was named astataricusone C.

**Figure 4:**
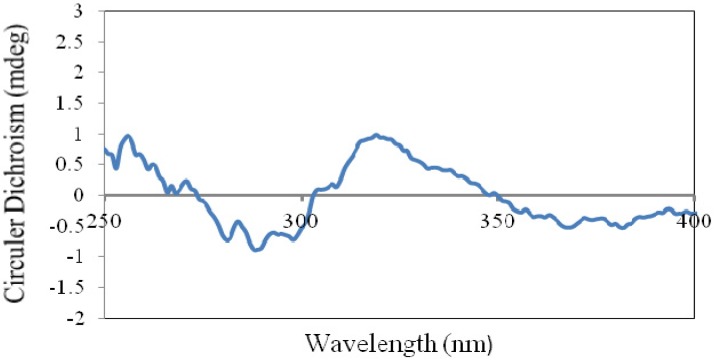
ICD spectrum of the *in situ*-formed Mo-complex of **4** and Mo_2_(OAc)_4_ in a ratio of 1:1.2.

Compound **4** was obtained as a white powder. Its molecular formula was determined as C_30_H_52_O_3_ by HREIMS at *m/z* 460.3922 [M]^+^ (calcd. for 460.3916). Comparing the NMR data with **1** indicated that **4** had a different side chain. The structure of **4** was established as 21,22-dihydroxy-shion-3-one based on the 1D and 2D NMR data. The stereochemistry at C-21 were amenable to CD analysis with dimolybdenum tetracetate. Acyclic 1,2-diols can form a complex with Mo_2_(AcO)_4_ and the complex produces a significant induced CD spectrum (ICD). According to the rule proposed by Snatzke [18,19], the diagnostic band at around 310 nm has the same sign of the O-C-C-O dihedral angle in the favored conformation in the 1,2-diol moiety. So assignment of the absolute configuration of the chiral centers in the 1,2-diol moiety could be given based on the sign of the band at arond 310 nm of the ICD spectrum. In particular an *S*-monosubstituted glycol gives rise to a positive Cotton effect at around 310 nm. Thus, the positive sign observed at 315 nm in the mixture ICD spectrum of **4** and Mo_2_(OAc)_4_ (Figure 4) allowed to assign the *S*-configuration to C-21 in **4**. Therefore, the structure of **4** was established as (4*R*,5*S*,8*S*,9*S*,10*S*,13*S*,14*R*,17*S*,21*S*)-21,22-dihydroxy-shion-3-one, and it was named astataricusone D.

Compound **5** was assigned the molecular formula C_30_H_52_O_2_ by its HREIMS data at *m/z* = 444.3973 [M]^+^ (calcd. 444.3967). Comparing the NMR data with **6** [14,15] and **1**, **5** had the same cyclic skeleton as **6** and the same side chain as **1**, which was supported by the HMBC correlations between H-21 (*δ*_H_ 3.99, 1H, t, *J* = 6.2 Hz) and C-29 (*δ*_C_ 17.3), C-20 (*δ*_C_ 29.8), C-19 (*δ*_C_ 38.9), C-30 (*δ*_C_ 111.2), and H-30 (*δ*_H_ 4.92, 1H, s) and C-21 (*δ*_C_ 77.0). The configuration of **5** was established to be identical to **1** based on the similar ROESY correlations and **6** based on the similar CD data. Therefore, the structure of **5** was established as (3*S*,4*R*,5*S*,8*S*,9*S*,10*S*,13*S*,14*R*,17*S*,21*S*)-shion-22(30)-en-3,21-diol, and the compound was named astataricusol A.

Compounds **1**–**6** were tested for anti-HBV activity in HBV antigen secretion and DNA replication of HepG 2.2.15 cells using the literature methods [20,21,22]. Results indicated that **2** showed inhibitory activity on HBsAg secretion with an IC_50_ value of 23.5 μM, while **2** and **6** showed inhibitory activities on HBeAg secretion with IC_50_ values of 18.6 and 40.5 μM, and cytotoxicity on HepG 2.2.15 cells with CC_50_ values of 172.4 and 137.7 μM, respectively; **2** and **6** exhibited inhibitory activities on HBV DNA replication with IC_50_ values of 2.7 and 30.7 μM.

## 3. Experimental

### 3.1. General

Melting points were determined on a TECH X-4 micro melting point apparatus without correction. Optical rotations were measured with a Horiba SEPA-300 polarimeter (Horiba, Kyoto, Japan). UV spectra were obtained using a Shimadzu UV-2401A spectrophotometer (Shimadzu, Kyoto, Japan). IR spectra were obtained by a Tenor 27 spectrophotometer (Bruker, Karlsruhe, Germany) using KBr pellets. CD spectra were recorded with an Applied Photophysics Chirascan spectrometer (Applied Photophysics Ltd., London, UK). 1D and 2D NMR spectra were run on Bruker DRX-500 or AM-400 spectrometers (Bruker, Karlsruhe, Germany) with TMS as internal standard; coupling constants were expressed in Hertz and chemical shifts (*δ*) were expressed in ppm with reference to the solvent signals. Mass spectra were recorded on a VG Autospec-3000 spectrometer (VG, Manchester, UK) or an API QSTAR Pulsar TOF spectrometer (AB-MDS Sciex, Concord, ON, Canada).

Analytical or semipreparative HPLC was performed on an Agilent 1100 liquid chromatograph (Agilent, Santa Clara, CA, USA) with a Zorbax Eclipse-C_18_ (4.6 mm × 150 mm; 9.4 mm × 250 mm; 5 μM). Column chromatography was performed on silica-gel (200–300 mesh, Qingdao Yu-Ming-Yuan Chemical Co. Ltd., Qingdao, China), Sephadex LH-20 (Pharmacia Fine Chemical Co., Uppsala, Sweden), Lichroprep RP−18 gel (40–63 μM, Merck, Darmstadt, Germany). Fractions were monitored by TLC (GF254, Qingdao Yu-Ming-Yuan Chemical Co. Ltd., Qingdao, China), and spots were visualized by heating Si gel plates sprayed with 5% H_2_SO_4_ in EtOH.

### 3.2. Plant Material

The roots and rhizomes of *Aster tataricus* L. f. was commercially purchased from the Yunnan Lv-Sheng Pharmaceutical Co. Ltd. (Kunming, China) and identified by Prof. Xi-Wen Li at Kunming Institute of Botany (voucher No. 200704).

### 3.3. *In Vitro* Anti-HBV Assay

The *in vitro* anti-HBV activity of compounds **1**–**6** were analyzed by the secretion of HBV antigen and DNA replication from the HepG 2.2.15 cell line. The toxicity of the compounds was evaluated by the sulforhodamine B method. DMSO alone was used as a solvent control. All the compounds were tested for their anti-HBV activity with the highest concentration of 20 μg/mL. Lamivudine (3TC) was used as the positive control.

#### 3.3.1. Cell Line and Cell Culture

The widely used HepG 2.2.15 cell line was applied for the assay of anti-HBV activity. In this study, the HepG 2.2.15 cell lines, which were stably transfected with the HBV genome, were a gift from Prof. Yong-Tang Zheng, Kunming Institute of Zoology, Chinese Academy of Sciences and cultured in RPMI−1640 (Gibco, Carlsbad, CA, USA) medium supplemented with 10% FBS (Haoyang Biological Manufacture Co., Ltd. Tianjin, China) and 400 μg/mL G148 (Calbiochem, San Diego, CA, USA). All cultures were maintained at 37 °C in a moist atmosphere containing 5% CO_2_.

#### 3.3.2. Analysis of Secreted HBV Antigens

Inhibitory activity of compounds **1**–**6** on the secretion of HBV antigens in HepG 2.2.15 cells was evaluated with an ELISA method (Kehua Bio-engineering Co., Ltd, Shanghai, China). The procedures were performed according to that described in the previous literature [20,21] with some modifications. Cells were seeded in 96-well microplates at a density of 5 × 10^4^ cells/mL and cultured at 37 °C, 5% CO_2_ for 24 h. Different concentrations of compounds were added in the wells. The medium was replaced every 3 days with fresh medium and compounds. After cultured for 12 days, the supernatants were collected, and the levels of HBsAg and HBeAg in the supernatants were evaluated according to the manufacturer’s instructions. The absorbance was measured at 450/630 nm using a microplate reader (SpectraMax 190, Molecular Devices, San Francisco, CA, USA).

#### 3.3.3. Assay for HBV DNA Replication

Inhibitory activity of compounds **2** and **6** on HBV DNA replication in HepG 2.2.15 cells was examined by a fluorescence quantitative PCR kit (Shanghai Fuxing Bio-engineering Co., Ltd, Shanghai, China). After the cells were treated with or without the compounds for 12 days, the HBV DNA level in HepG2.2.15 cells was evaluated according to the manufacturer’s instruction with a Real-Time PCR Systems (ABI7500, Applied Biosystems, Foster city, CA, USA).

#### 3.3.4. Cytotoxicity Assay

Cytotoxicity of compounds **1**–**6** on HepG 2.2.15 cells was tested by a sulforhodamine B method [22] (SRB, Sigma, St. Louis, MO, USA). Firstly, HepG 2.2.15 cells were seeded in a 96-well microplate for 24 h. Compounds, dissolved in DMSO and diluted with the medium, were placed in each well and incubated for another 72 h at 37 °C. Then cells were fixed with 50% ice-cold trichloroacetic acid at 4 °C for 1 h and stained with 0.4% SRB in 1% acetic acid solution for 15 min. After removal of excessive dye, SRB was resuspended in 10 mM Tris buffer, and the absorbance was measured at 560 nm with the microplate reader above-mentioned.

### 3.4. Extraction and Isolation

The air-dried roots and rhizomes (50 kg) of *Aster tataricus* were extracted with methanol at reflux for three times. The MeOH extract (13 kg) was concentrated under reduced pressure, and then partitioned sequentially by EtOAc and *n*-BuOH. The dried EtOAc part (2 kg) was subjected to silica gel column chromatography (CC, 10 kg) and eluted by a gradient of CHCl_3_/MeOH. The fraction from CHCl_3_ (840 g) was subjected to silica gel CC (1:0–5:1 petroleum ether/acetone), giving fractions (Fr.) 1–6. Fr. 4 (40 g, 20:1 petroleum ether/acetone) was resubmitted to chromatography over silica gel (30:1–10:1 petroleum ether/acetone) to yield Fr. 4/1–4/3. Fr. 4/2 (20:1 petroleum ether/acetone, 25 g) was purified by Sephadex LH-20 (1:1 chloroform/methanol) and then submitted to additional silica gel CC (20:1, 10:1, and 5:1 petroleum ether/ethyl acetate) to afford Fr. 4/2/1–4/2/3. Fr. 4/2/2 (10:1 petroleum ether/ethyl acetate, 10 g) was purified by CC on RP−18 with MeOH–H_2_O (40:60–1:0) to yield Fr. 4/2/2/1–4/2/2/4. Fr. 4/2/2/3 (1.55 g) was purified by semipreparative HPLC with MeCN–H_2_O (75:25) at a flow rate of 10 mL/min (UV detector, 205 nm) to yield compounds **1** (t_R_ = 15.5 min, 152 mg), **2** (t_R_ = 16.4 min, 121 mg), **3** (t_R_ = 17.5 min, 45 mg) and **6** (t_R_ = 21.0 min, 75 mg). Fr. 4/3 (10:1 petroleum ether/acetone, 8 g) was purified by CC on RP−18 (MeOH–H_2_O, 30:70–1:0) to yield Fr. 4/3/1–4/3/4. Compounds **4** (t_R_ = 17.1 min, 5 mg) and **5** (t_R_ = 23.0 min, 7 mg) were obtained from Fr. 4/3/3 by subsequent silica gel CC (petroleum ether/acetone, 15:1) and semipreparative HPLC with MeCN–H_2_O (70:30) at a flow rate of 10 mL/min (UV detector, 205 nm).

*Astataricusone A* (**1**): colorless needles; mp 235–236 °C; 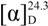 −33.8 (c 1.33, MeOH); UV (MeOH) λ_max_ (logε) 202 (3.12) nm; CD (c 0.64, MeOH) λ (Δε) 290 (−9.5) nm; IR (KBr) ν_max_ 3,498, 2,955, 2,927, 1,697, 1,467, 1,451, 1,390, 1,074, 894 cm^–1^; ^1^H-NMR (400 MHz, CDCl_3_) and ^13^C-NMR (100 MHz, CDCl_3_), see Table 1 and Table 2; HREIMS *m/z* 442.3808 [M]^+^ (calcd. for C_30_H_50_O_2_, 442.3811).

*Astataricusone B* (**2**): white powder; 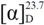 −5.9 (c 0.27, MeOH); UV (MeOH) λ_max_ (log ε) 209 (2.87) nm; CD (c 0.61, MeOH) λ (Δε) 290 (−2.5) nm; IR (KBr) ν_max_ 3,441, 3,425, 2,961, 2,926, 1,704, 1,629, 1,262, 1,097, 1,024, 803 cm^–1^; ^1^H-NMR (400 MHz, CDCl_3_) and ^13^C-NMR (100 MHz, CDCl_3_), see Table 1 and Table 2; HREIMS *m/z* 442.3804 [M]^+^ (calcd. for C_30_H_50_O_2_, 442.3811).

*Astataricusone C* (**3**): white powder; 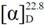 −18.6 (c 0.80, MeOH); UV (MeOH) λ_max_ (logε) 202 (3.18) nm; CD (c 0.64, MeOH) λ (Δε) 290 (−7.8) nm; IR (KBr) ν_max_ 3,533, 3,448, 2,933, 2,876, 1,711, 1,466, 1,387, 1,187, 972 cm^–1^; ^1^H-NMR (400 MHz, CDCl_3_) and ^13^C-NMR (100 MHz, CDCl_3_), see Table 1 and Table 2; HREIMS *m/z* 442.3824 [M]^+^ (calcd. for C_30_H_50_O_2_, 442.3811).

*Astataricusone D* (**4**): white powder; 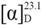 −98.1 (c 0.21, MeOH); UV (MeOH) λ_max_ (logε) 204 (3.41) nm; CD (c 0.60, DMSO) λ (Δε) 290 (−5.8), 300 (−5.7) nm; IR (KBr) ν_max_ 3,475, 3,443, 2,934, 1,701, 1,466, 1,388, 1,072 cm^–1^; ^1^H-NMR (400 MHz, CDCl_3_) and ^13^C-NMR (100 MHz, CDCl_3_), see Table 1 and Table 2; HREIMS *m/z* 460.3922 [M]^+^ (calcd. for C_30_H_52_O_3_, 460.3916).

*Astataricusol A* (**5**): white powder; 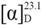 −19.0 (c 0.25, MeOH); UV (MeOH) λ_max_ (logε) 193 (2.56), 202 (2.99) nm; CD (c 0.63, MeOH) λ (Δε) 290 (−0.8) nm; IR (KBr) ν_max_ 3,439, 2,941, 2,925, 1,631, 1,463, 1,376 cm^–1^; ^1^H-NMR (400 MHz, CDCl_3_) and ^13^C-NMR (100 MHz, CDCl_3_), see Table 1 and Table 2; HREIMS *m/z* 444.3973 [M]^+^ (calcd. for C_30_H_52_O_2_, 444.3967).

### 3.5. X-ray Crystallographic Analysis

The intensity data for astataricusone A (**1**) was collected on a Bruker APEX DUO diffractometer using graphitemonochromated CuKα radiation. Its structure was solved by direct methods (SHELXS97), expanded using difference Founier techniques, and refined by the program and full-matrix least-squares calculations. The nonhydrogen atoms were refined anisotropically, and hydrogen atoms were fixed at calculated positions. Crystallographic data for the structure of **1** has been deposited in the Cambridge Crystallographic Data Centre (deposition number CCDC 926578). Copies of the data can be obtained free of charge from the CCDC via www.ccdc.cam.ac.uk. C_30_H_50_O_2_, *M* = 442.70, orthorhombic, *a* = 15.1404 (**2**) Å, *b* = 23.8310 (**3**) Å, *c* = 7.25500 (**10**) Å, *α* = 90.00°, *β* = 90.00°, *γ* = 90.00°, *V* = 2617.68 (**6**) Å^3^, *T* = 100(**2**) K, space group *P*2_1_2_1_2_1_, *Z* = 4, *μ*(CuKα) = 0.510 mm^−1^, 12841 reflections measured, 4644 independent reflections (*R_int_* = 0.0414). The final *R_1_* values were 0.0499 (*I* > 2*σ*(*I*)). The final *wR*(*F*^2^) values were 0.1382 (*I* > 2*σ*(*I*)). The final *R_1_* values were 0.0505 (all data). The final *wR*(*F*^2^) values were 0.1392 (all data). The goodness of fit on *F*^2^ was 1.064. Flack parameter = 0.1 (**3**). The Hooft parameter is 0.06 (**9**) for 1887 Bijvoet pairs.

### 3.6. Determination of Absolute Conﬁguration of the 21,22-Diol Group in **4**

According to the procedure [18], the first CD spectrum (CD_1_) of the 1:1.2 mixture of **4**:Mo_2_(OAc)_4_ at the concentration of 0.95 mg/mL in DMSO was recorded after mixed immediately. Then the second CD spectrum (CD_2_) was recorded at 30 min after mixed. Finally the induced CD (ICD) of **4** was calculated by the formula: ICD = CD_2_ − CD_1_.

## 4. Conclusions

Five new shionane-type triterpenes **1**–**5**, named astataricusones A–D (compound **1**–**4**) and astataricusol A (**5**), were isolated from *Aster tataricus*, together with a known one **6**. According to previous investigations on *Aster* species, we have evaluated the inhibitory activities of all compounds against the secretion of HBV antigen and DNA replication from the HepG 2.2.15 cell line. Compounds **2** and **6** showed significant anti-HBV activity. The results prove the potential for the use of this plant as a herbal medicine in the treatment of HBV.

## Supplementary Materials

^1^H-NMR, ^13^C-NMR and MS spectra of **1**–**5**, CD of **1**–**6**, and X-ray crystallographic data for **1** are available as supporting data, which can be accessed at: http://www.mdpi.com/1420-3049/18/12/14585/s1.
