# Supplementary material for: Astataricusones A–D and Astataricusol A, Five New Anti-HBV Shionane-Type Triterpenes from Aster tataricus L. f
Source: Molecules. 2013 Nov 25;18(12):14585–96. doi: 10.3390/molecules181214585 (PMC6270206; doi:10.3390/molecules181214585)

Supporting Information

Figure S1. ^{1}H-NMR ($CDCl_3$, 400 MHz) spectrum of Astataricusone A (**1**).

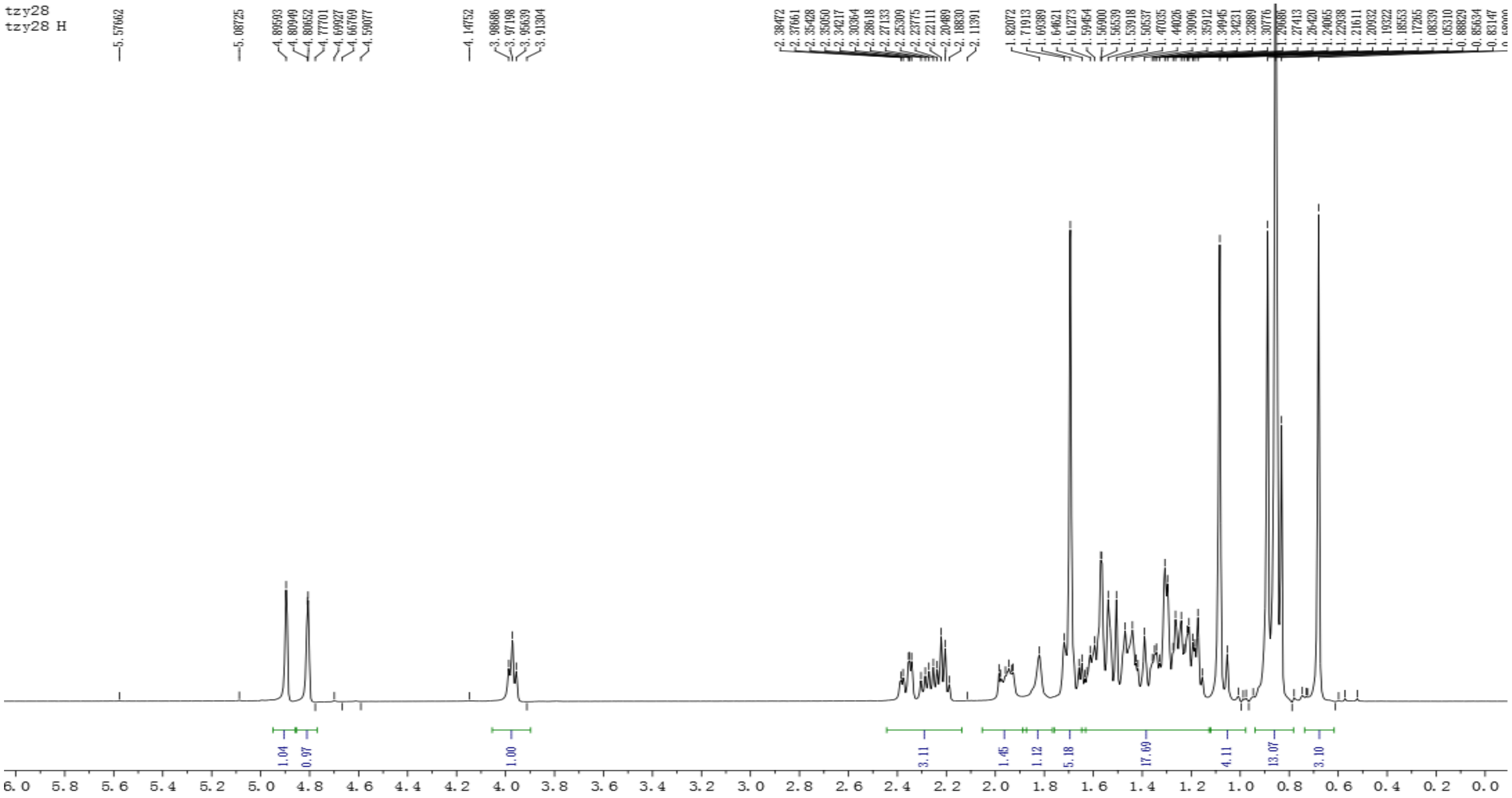

Figure S2. ^{13}C-NMR ($CDCl_3$, 100 MHz) spectrum of Astataricusone A (**1**).

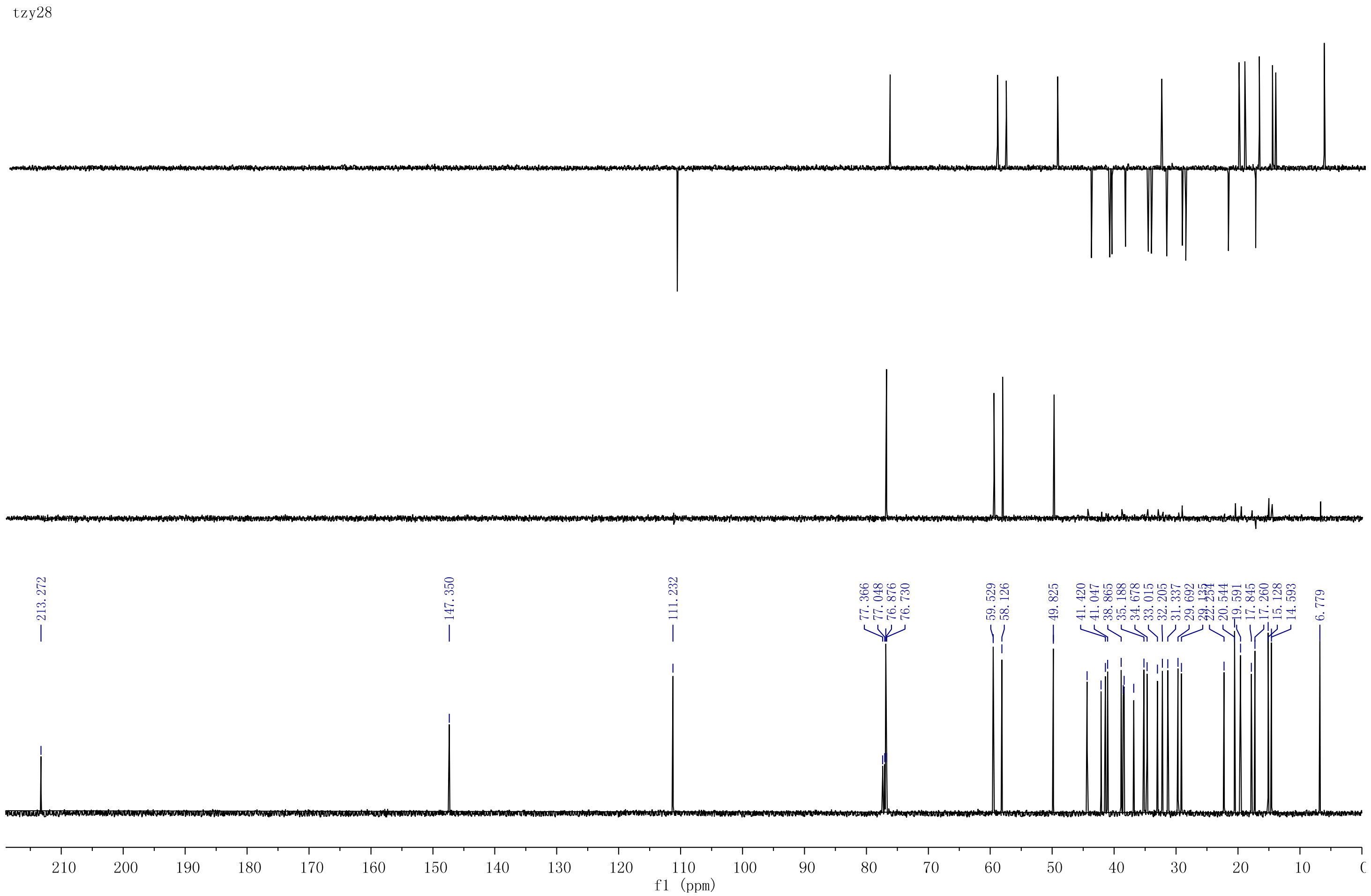

Figure S3. HSQC ($CDCl_3$, 500 MHz) spectrum of Astataricusone A (**1**).

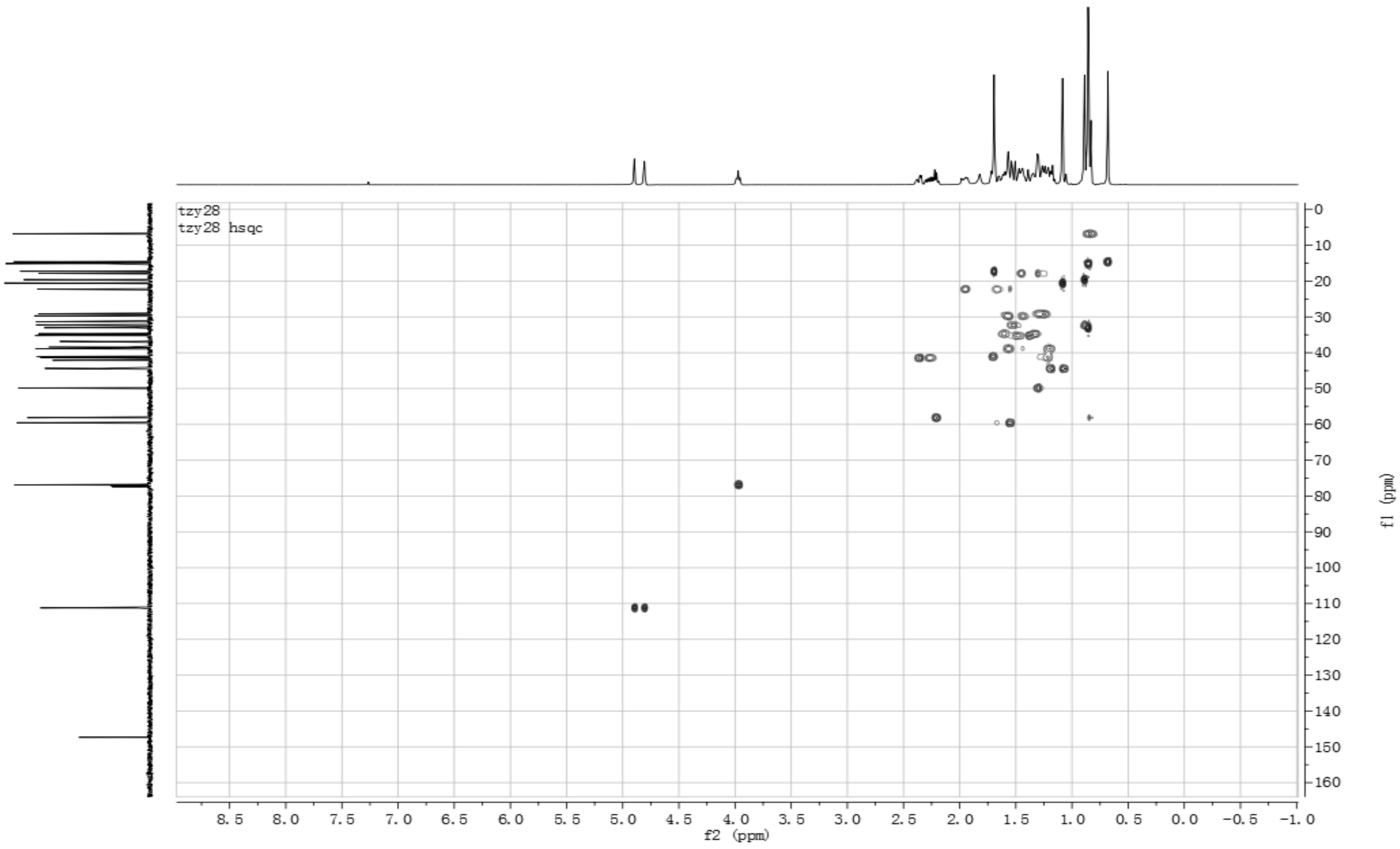

Figure S4. ^{1}H-^{1}H COSY ($CDCl_3$, 500 MHz) spectrum of Astataricusone A (**1**).

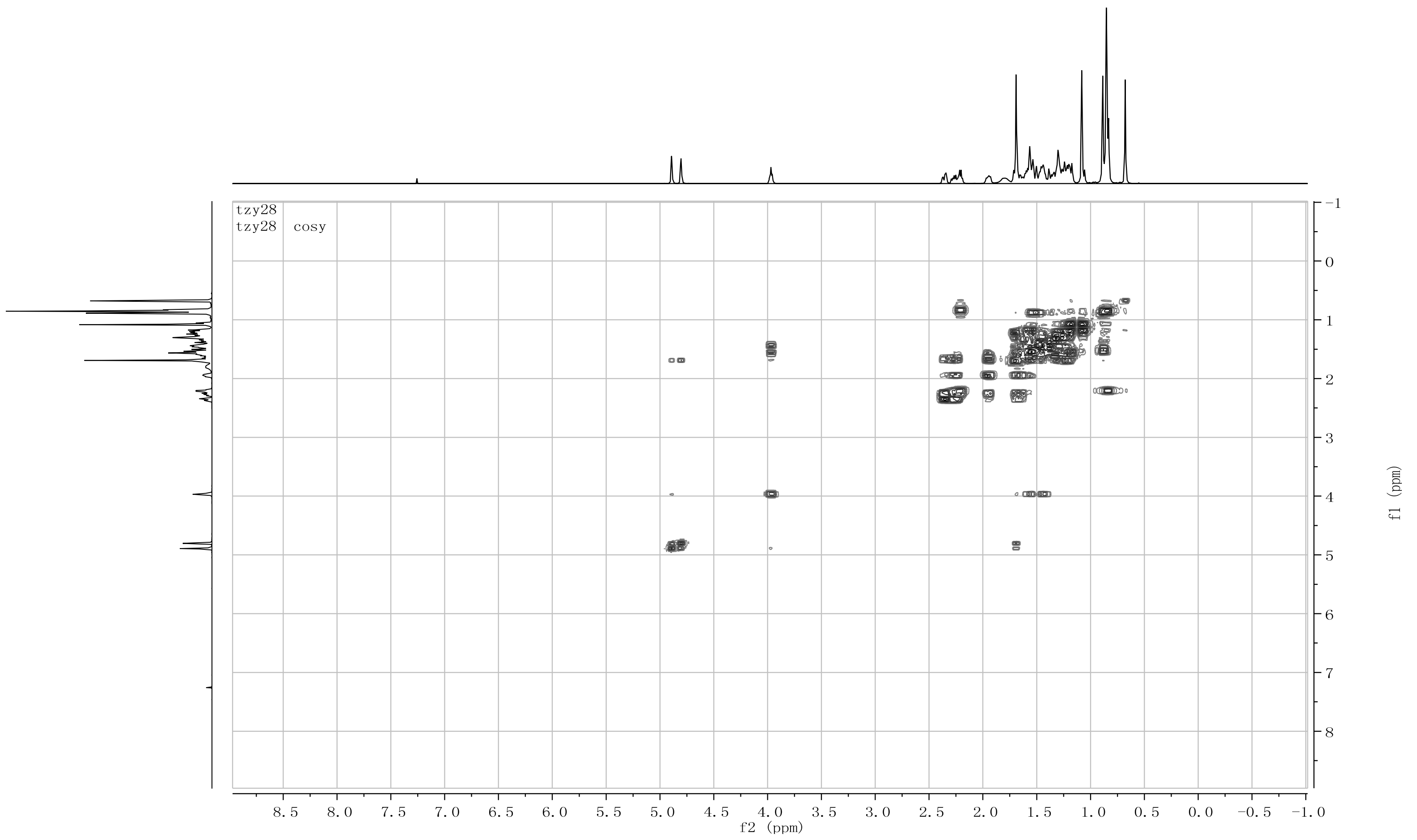

Figure S5. HMBC ($CDCl_3$, 500 MHz) spectrum of Astataricusone A (**1**).

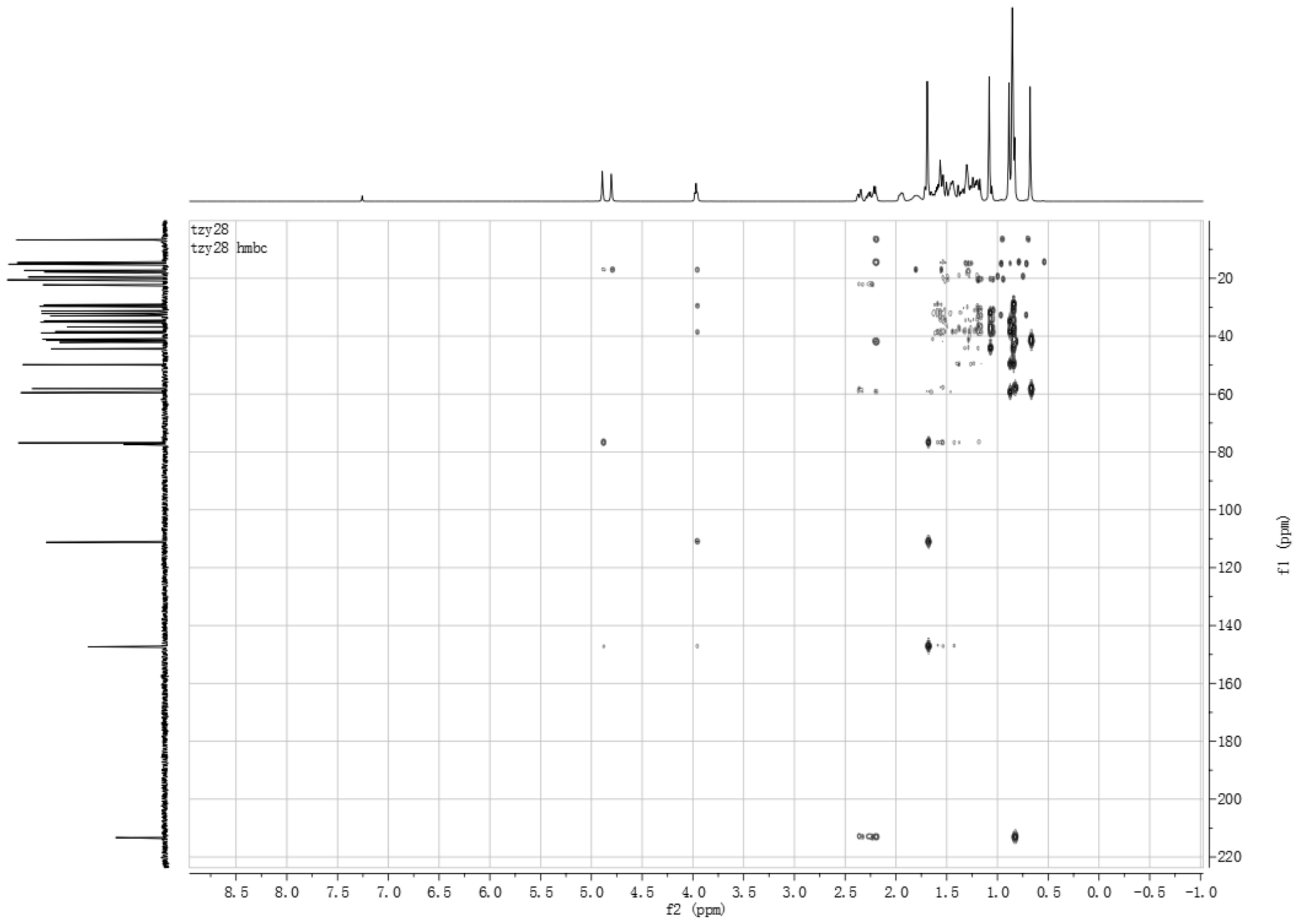

Figure S6. ROESY ($CDCl_3$, 500 MHz) spectrum of Astataricusone A (**1**).

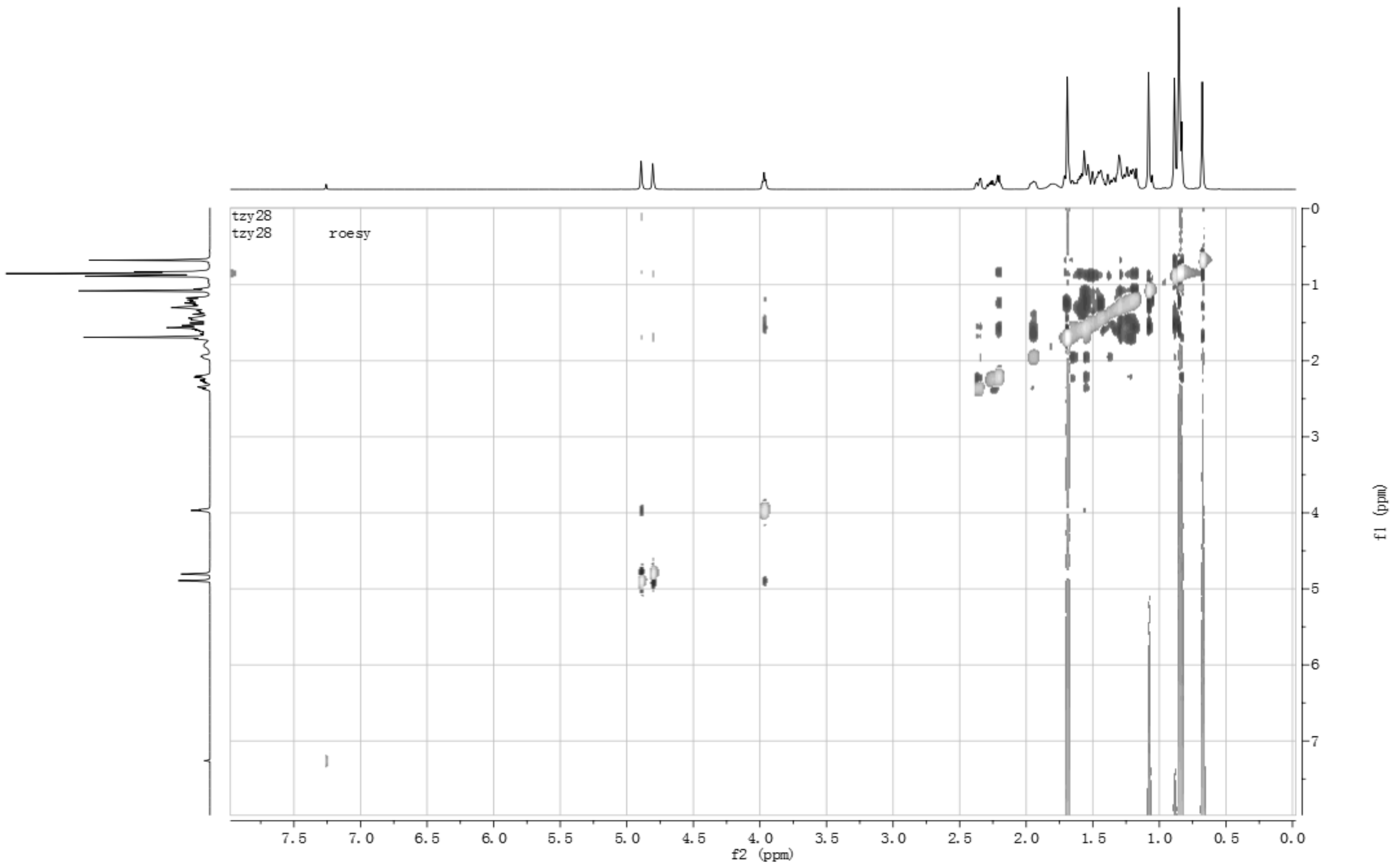

Figure S7. HREIMS spectrum of Astataricusone A (**1**).

Elemental Composition Report **Page 1**

Single Mass Analysis
Tolerance = 10.0 PPM / DBE: min = -10.0, max = 120.0
Selected filters: None

Monoisotopic Mass, Odd and Even Electron Ions
27 formula(e) evaluated with 1 results within limits (up to 51 closest results for each mass)
Elements Used:
C: 0-200 H: 0-400 O: 0-3

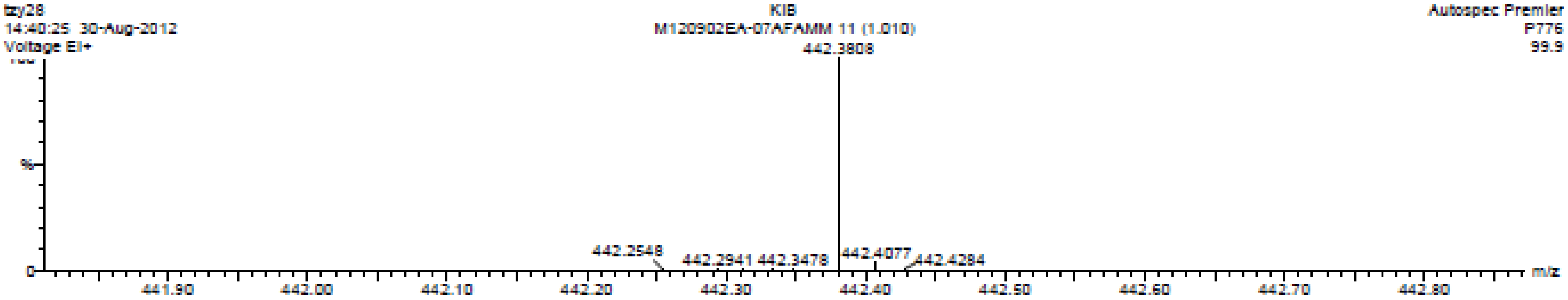

Minimum:				-10.0		
Maximum:		100.0	10.0	120.0		
Mass	Calc. Mass	mDa	PPM	DBE	i-FIT	Formula
442.3808	442.3811	-0.3	-0.7	6.0	5546068.5	C30 H50 O2

Figure S8. CD (MeOH) spectrum of Astataricusone A (**1**).

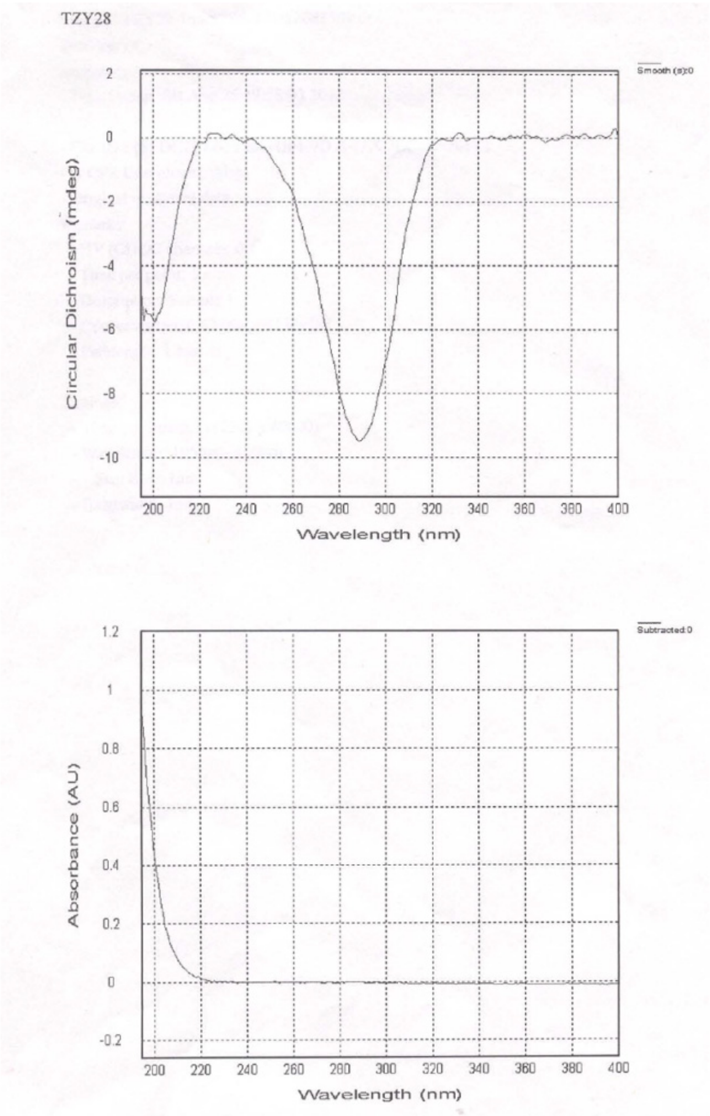

Figure S9. X-ray crystallographic structure of Astataricusone A (**1**).

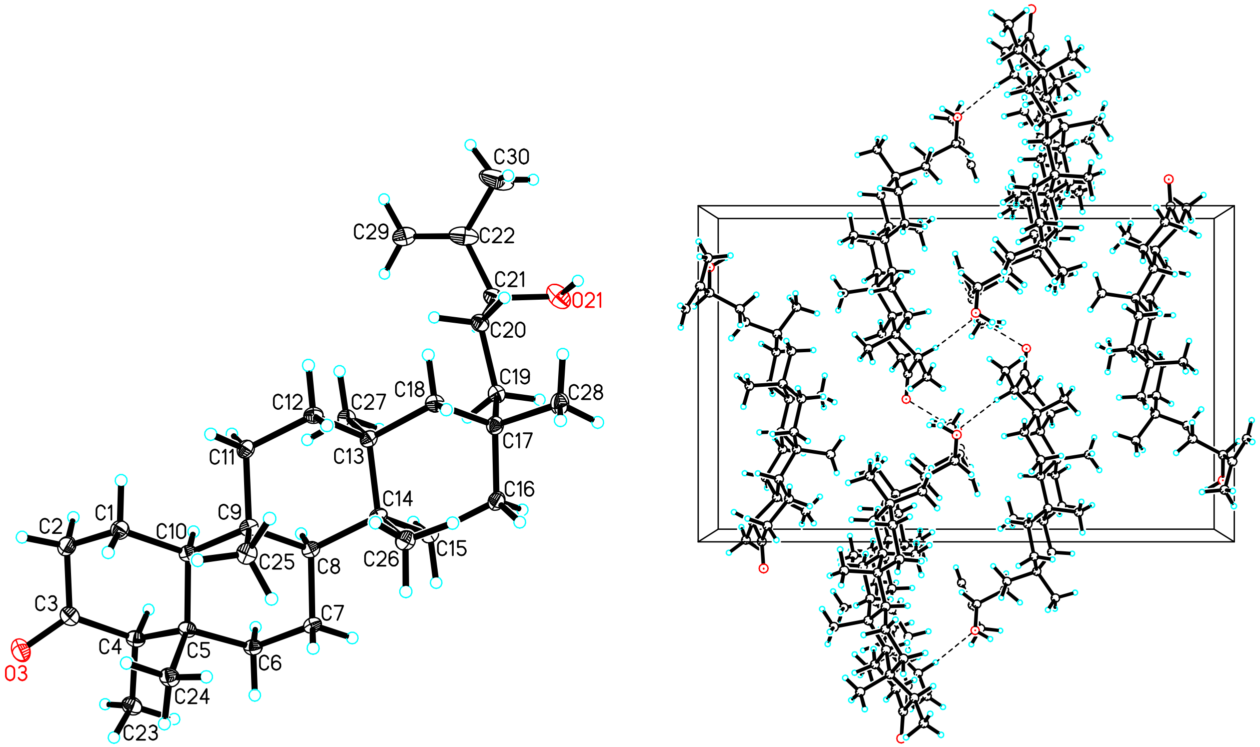

Figure S10. ^{1}H-NMR ($CDCl_3$, 400 MHz) spectrum of Astataricusone B (**2**).

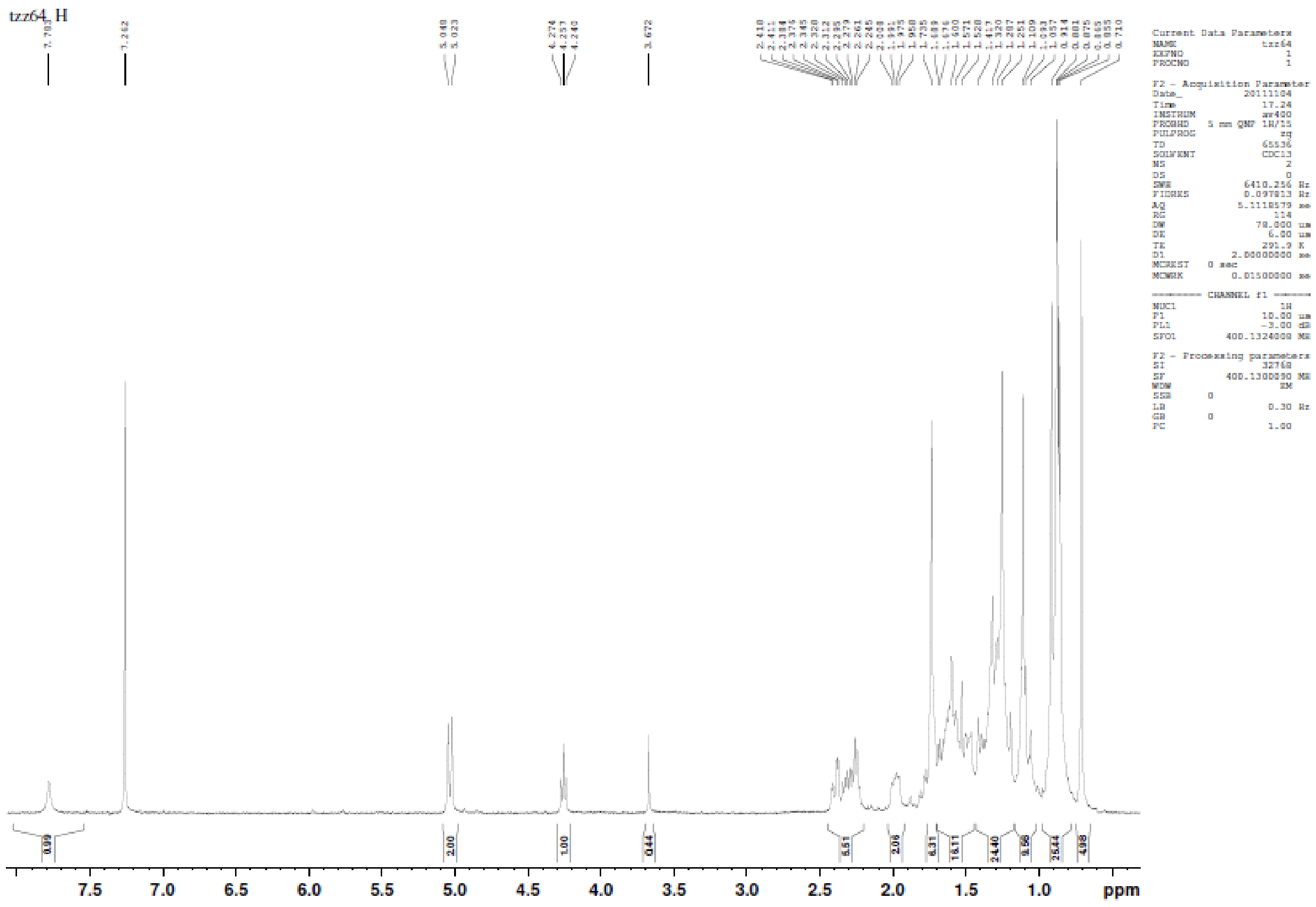

Figure S11. ^{13}C-NMR ($CDCl_3$, 100 MHz) spectrum of Astataricusone B (**2**).

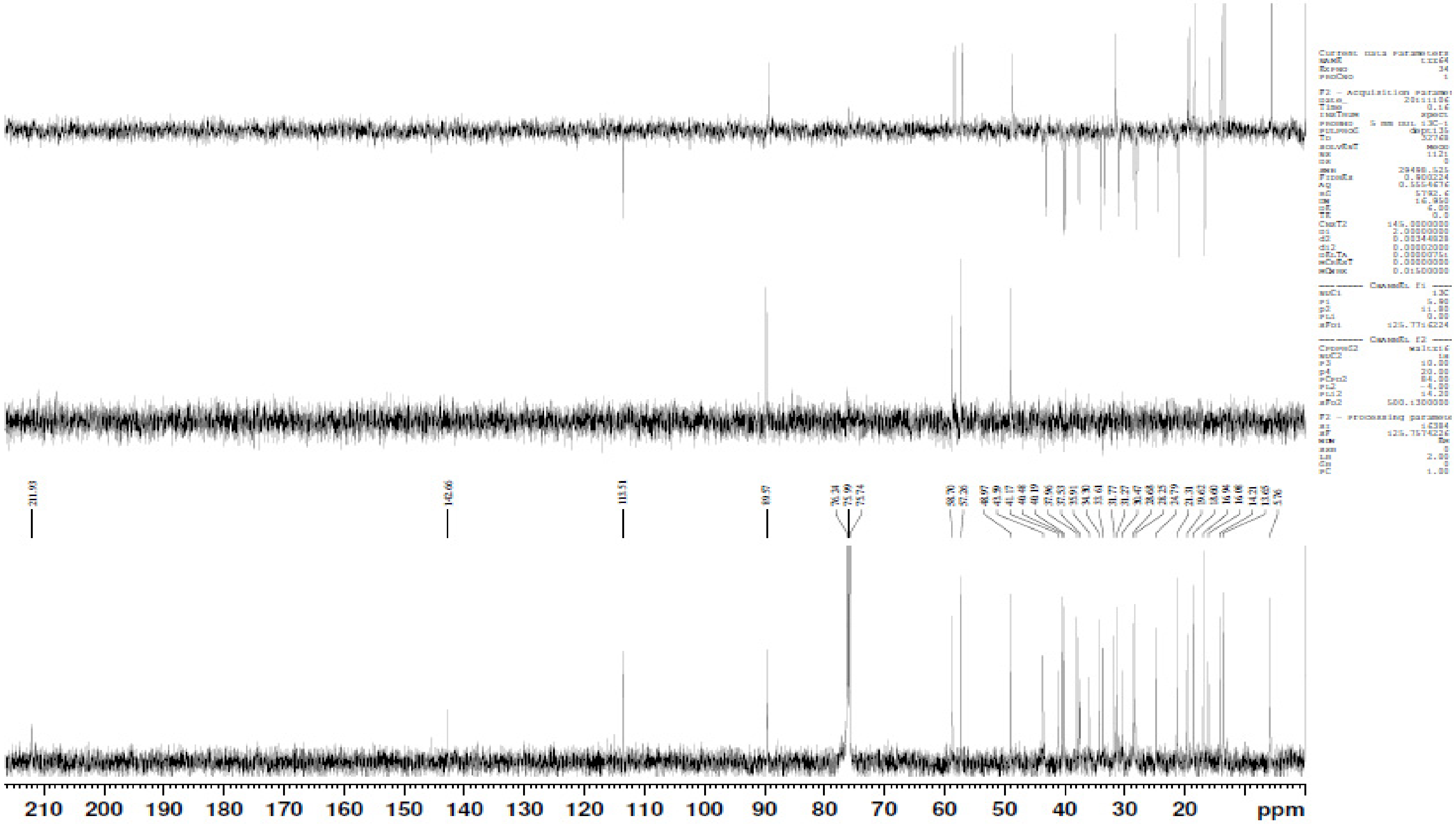

Figure S12. HSQC ($CDCl_3$, 500 MHz) spectrum of Astataricusone B (**2**).

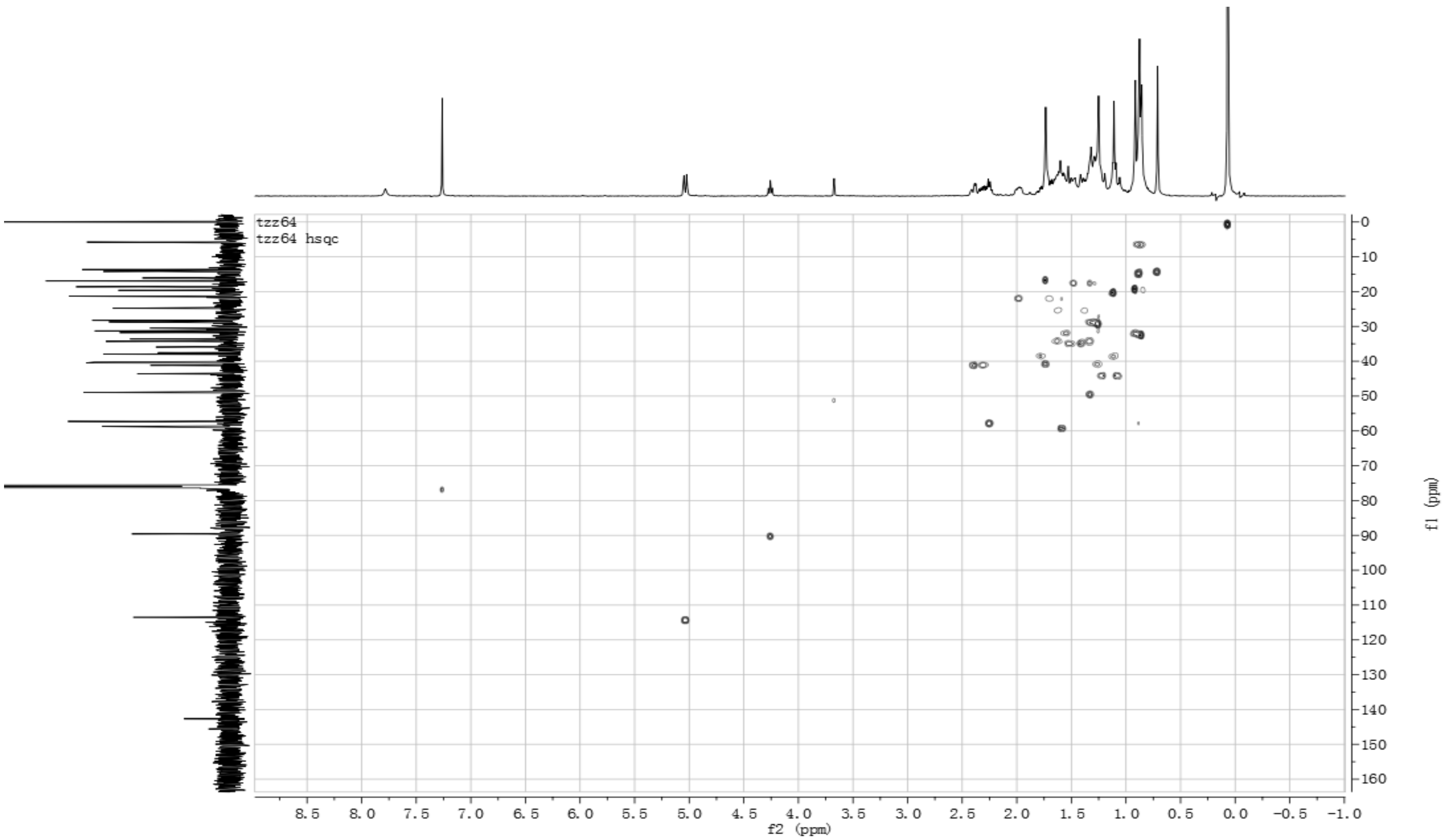

Figure S13. ¹H-¹H COSY ($CDCl_3$, 500 MHz) spectrum of Astataricusone B (**2**).

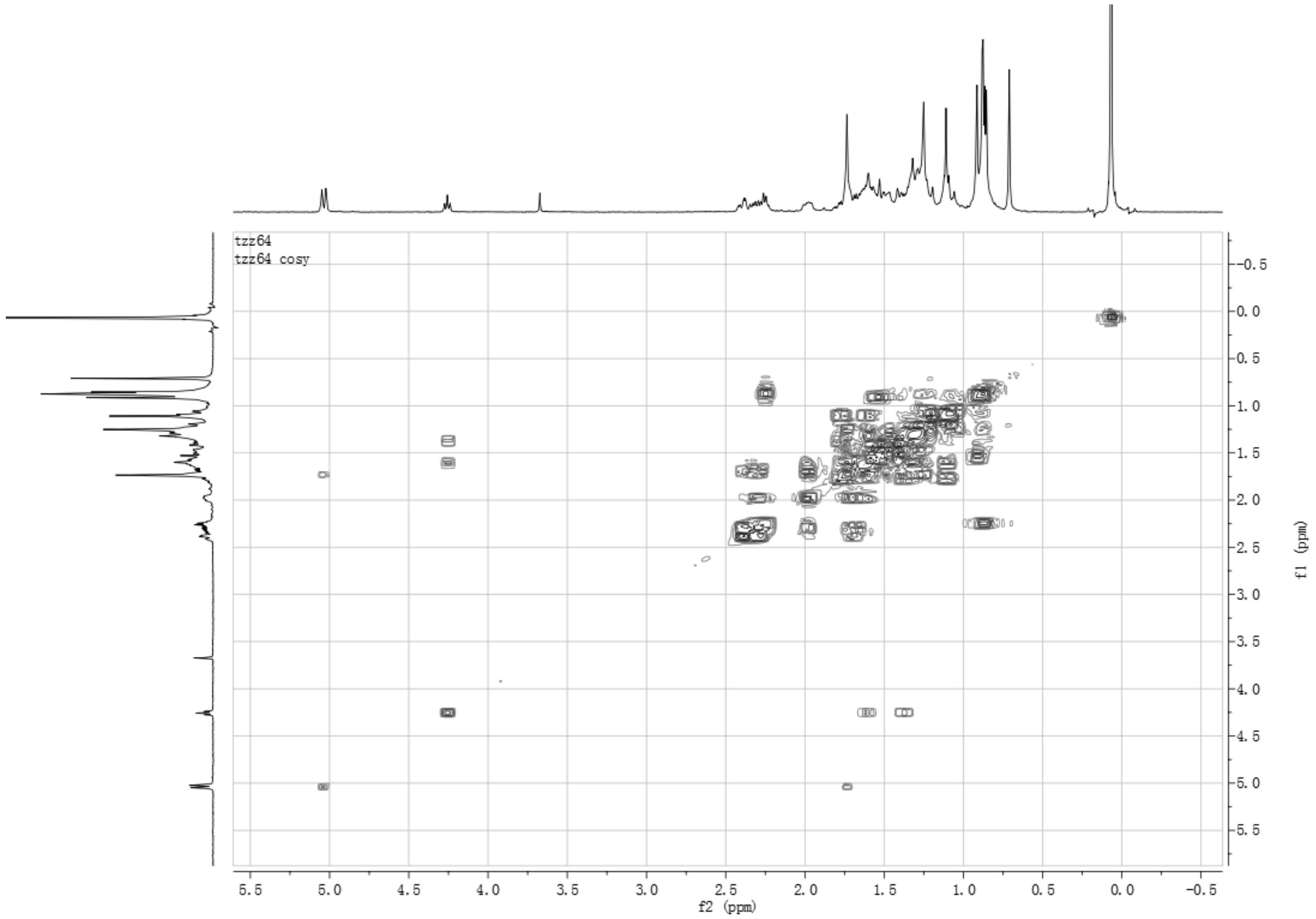

Figure S14. HMBC ($CDCl_3$, 500 MHz) spectrum of Astataricusone B (**2**).

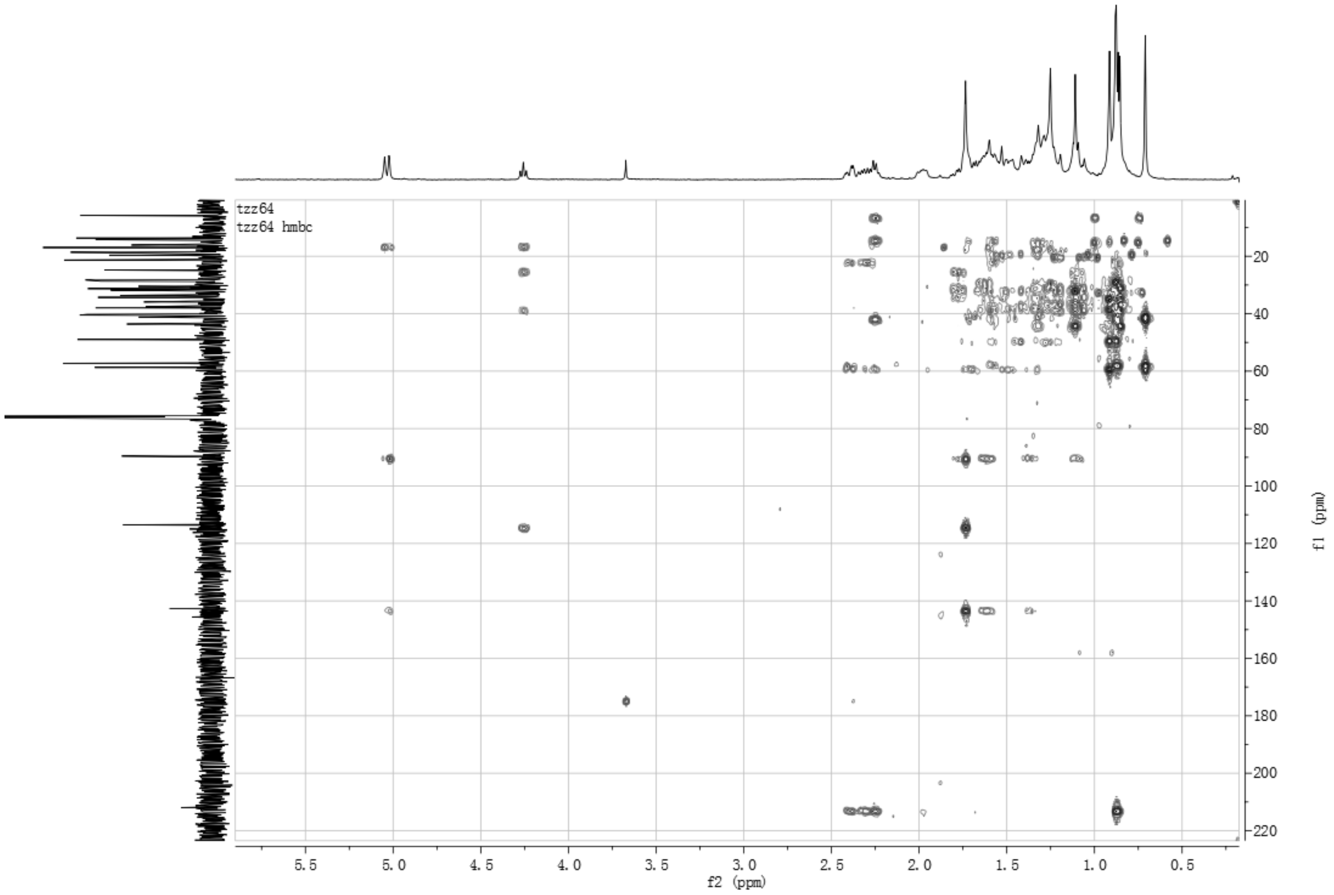

Figure S15. ROESY ($CDCl_3$, 500 MHz) spectrum of Astataricusone B (**2**).

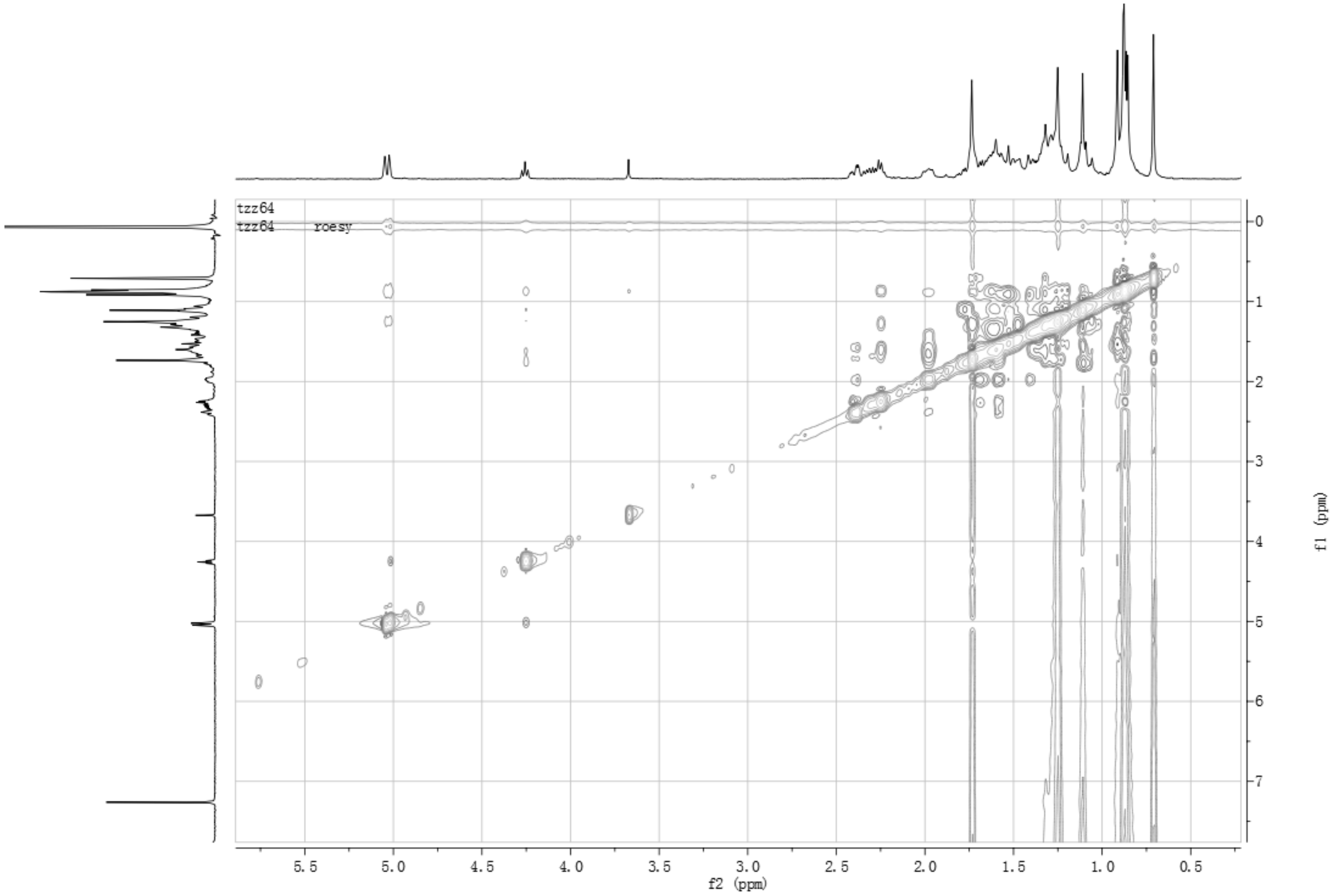

Figure S16. HREIMS spectrum of Astataricusone B (**2**).

Elemental Composition Report Page 1

Single Mass Analysis
Tolerance = 10.0 PPM / DBE: min = -10.0, max = 120.0
Selected filters: None

Monoisotopic Mass, Odd and Even Electron Ions
27 formula(e) evaluated with 1 results within limits (up to 51 closest results for each mass)
Elements Used:
C: 0-200 H: 0-400 O: 0-3

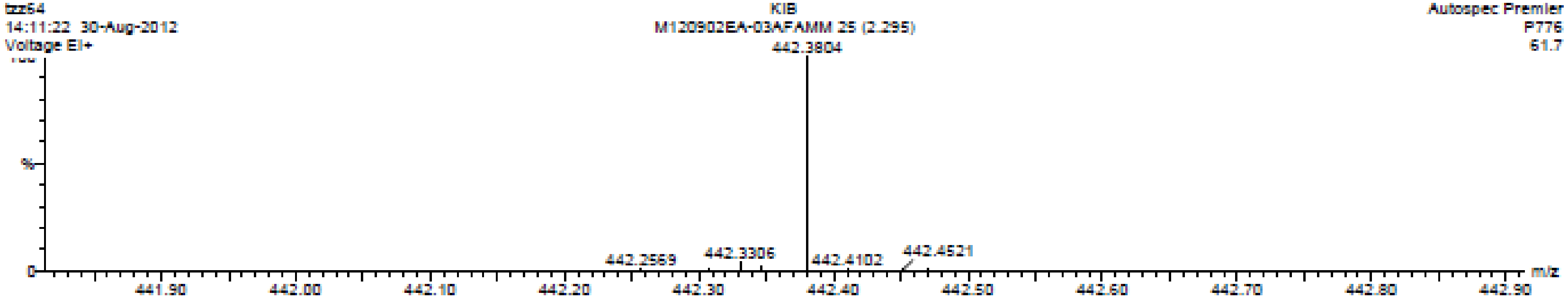

Minimum:				-10.0		
Maximum:		100.0	10.0	120.0		
Mass	Calc. Mass	mDa	PPM	DBE	i-FIT	Formula
442.3804	442.3811	-0.7	-1.6	6.0	5546049.5	C30 H50 O2

Figure S17. CD (MeOH) spectrum of Astataricusone B (**2**).

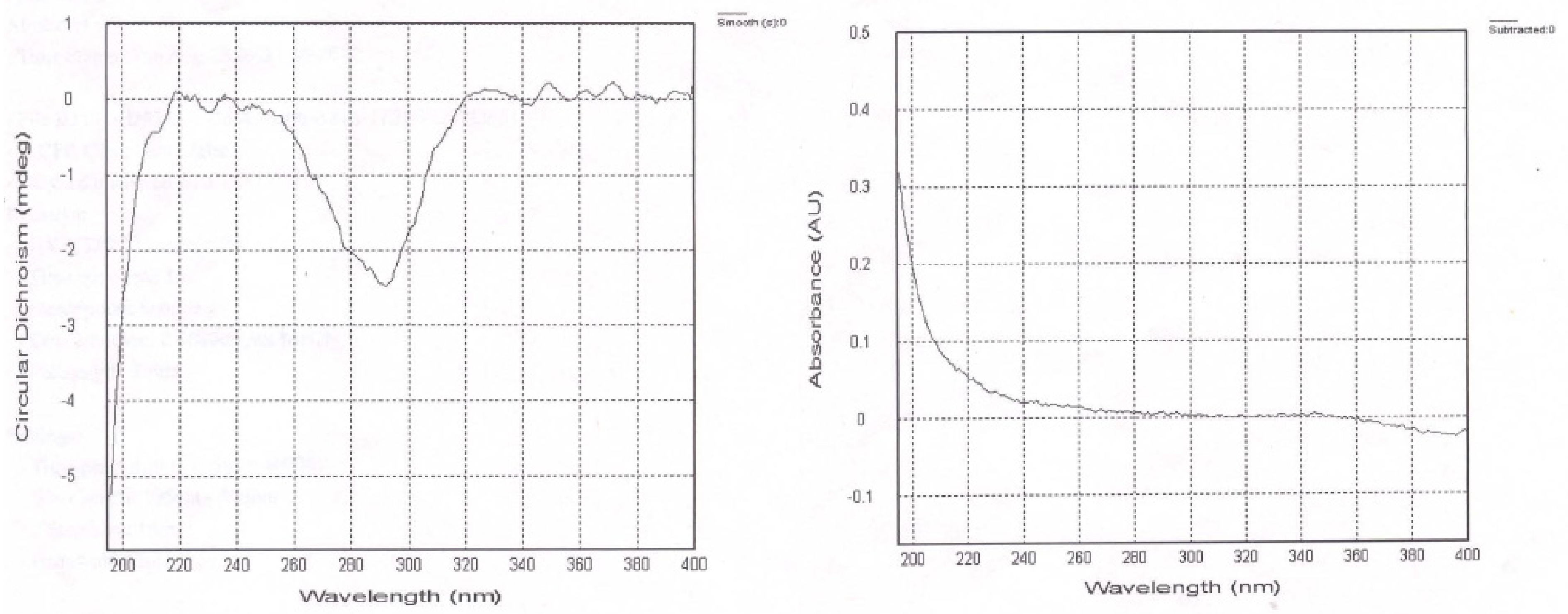

Figure S18. ^{1}H NMR ($CDCl_3$, 400 MHz) spectrum of Astataricusone C (**3**).

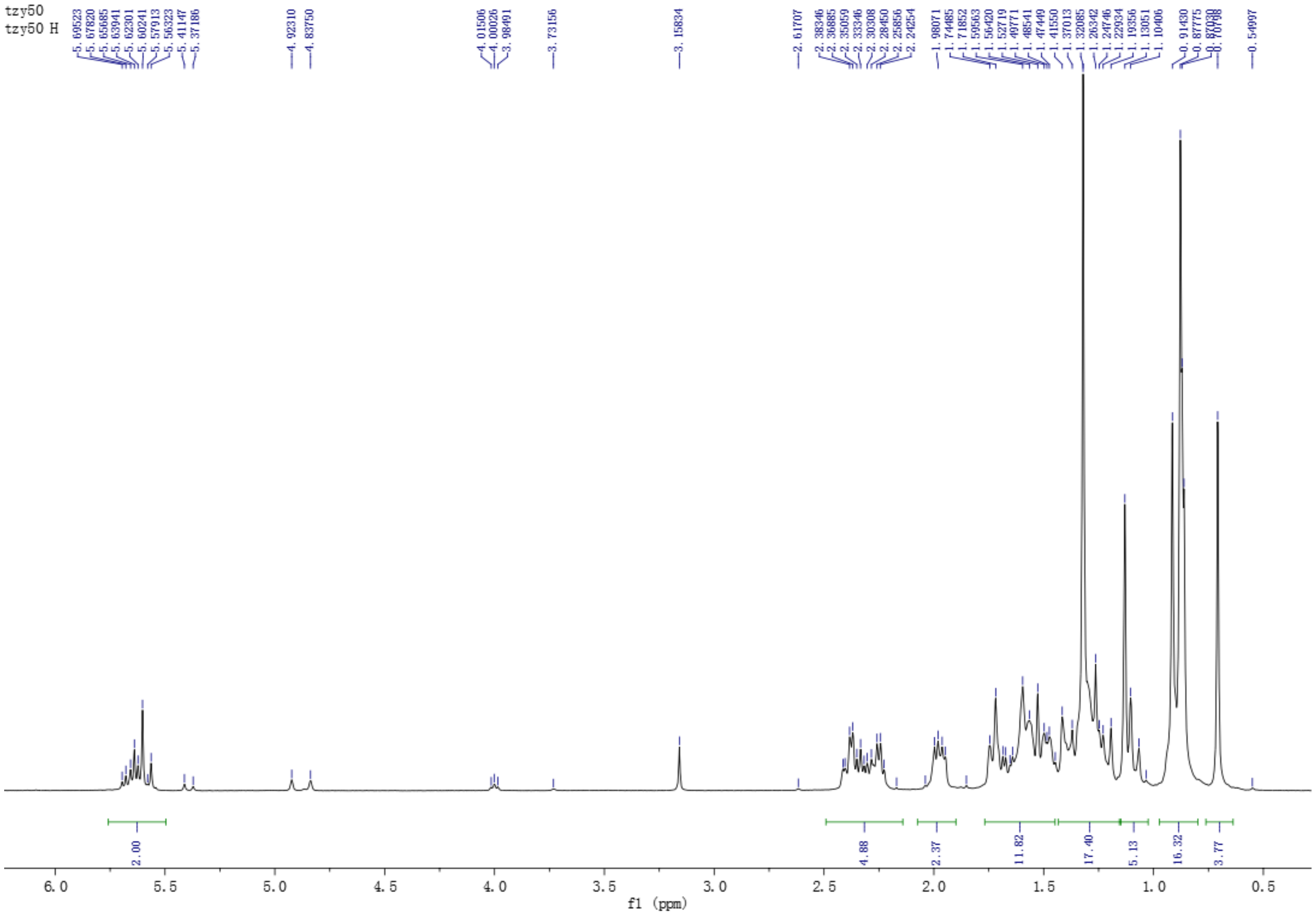

Figure S19. ^{13}C NMR ($CDCl_3$, 100 MHz) spectrum of Astataricusone C (**3**).

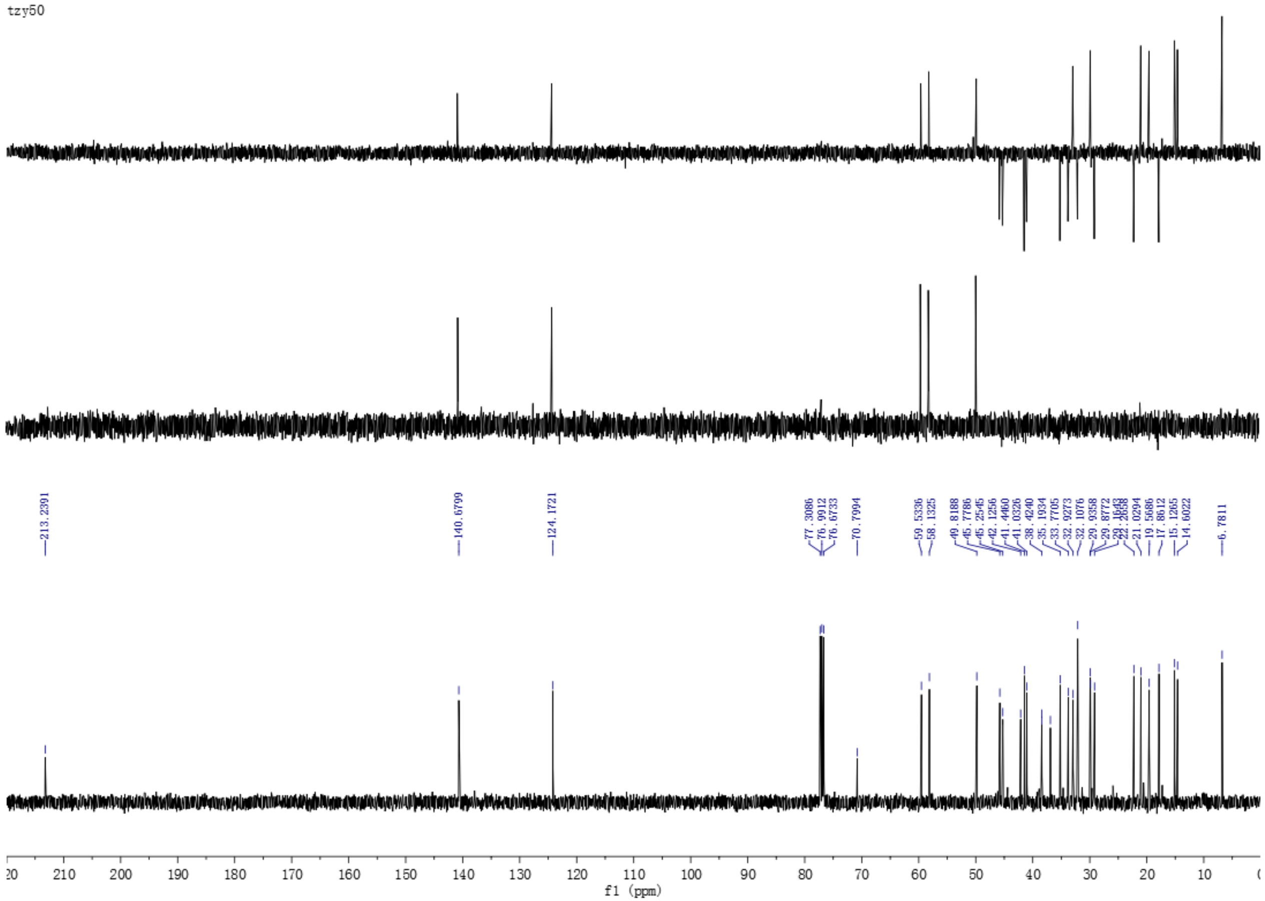

Figure S20. HSQC ($CDCl_3$, 500 MHz) spectrum of Astataricusone C (**3**).

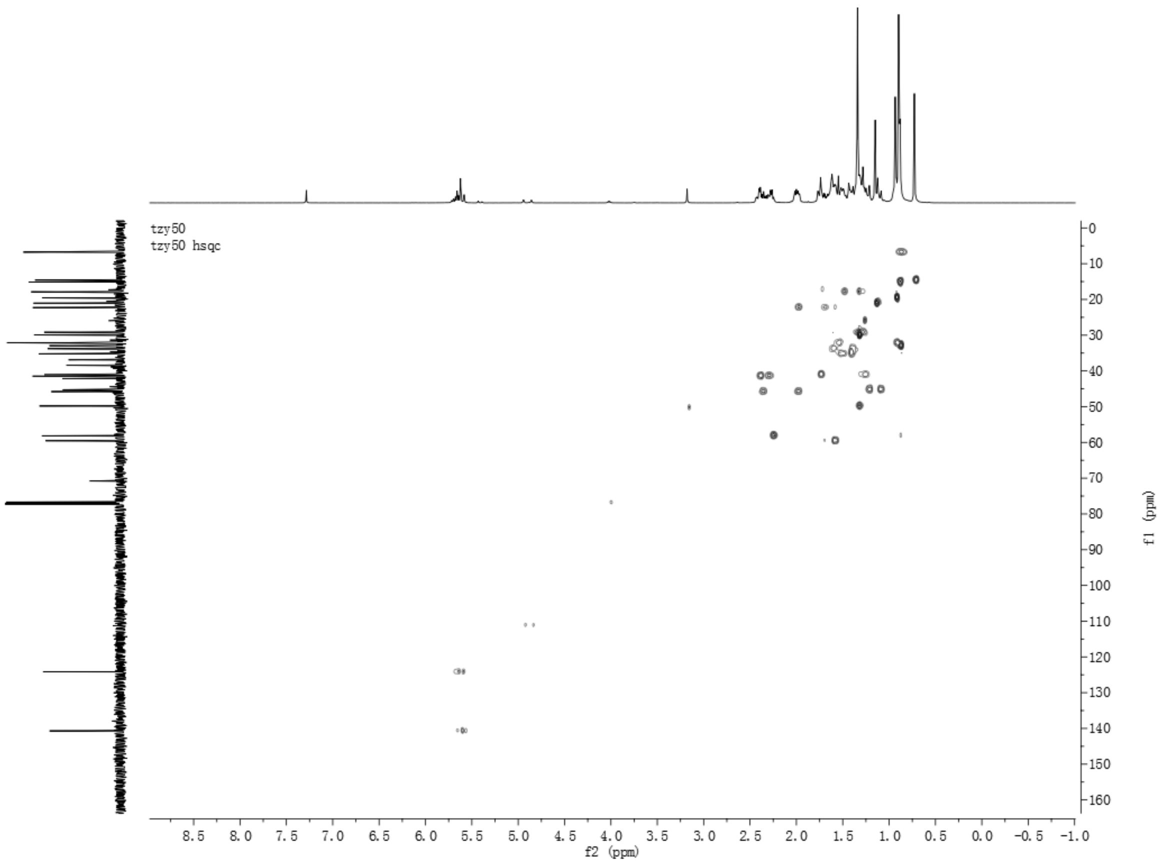

Figure S21. ^1H-^1H COSY ($CDCl_3$, 500 MHz) spectrum of Astataricusone C (**3**).

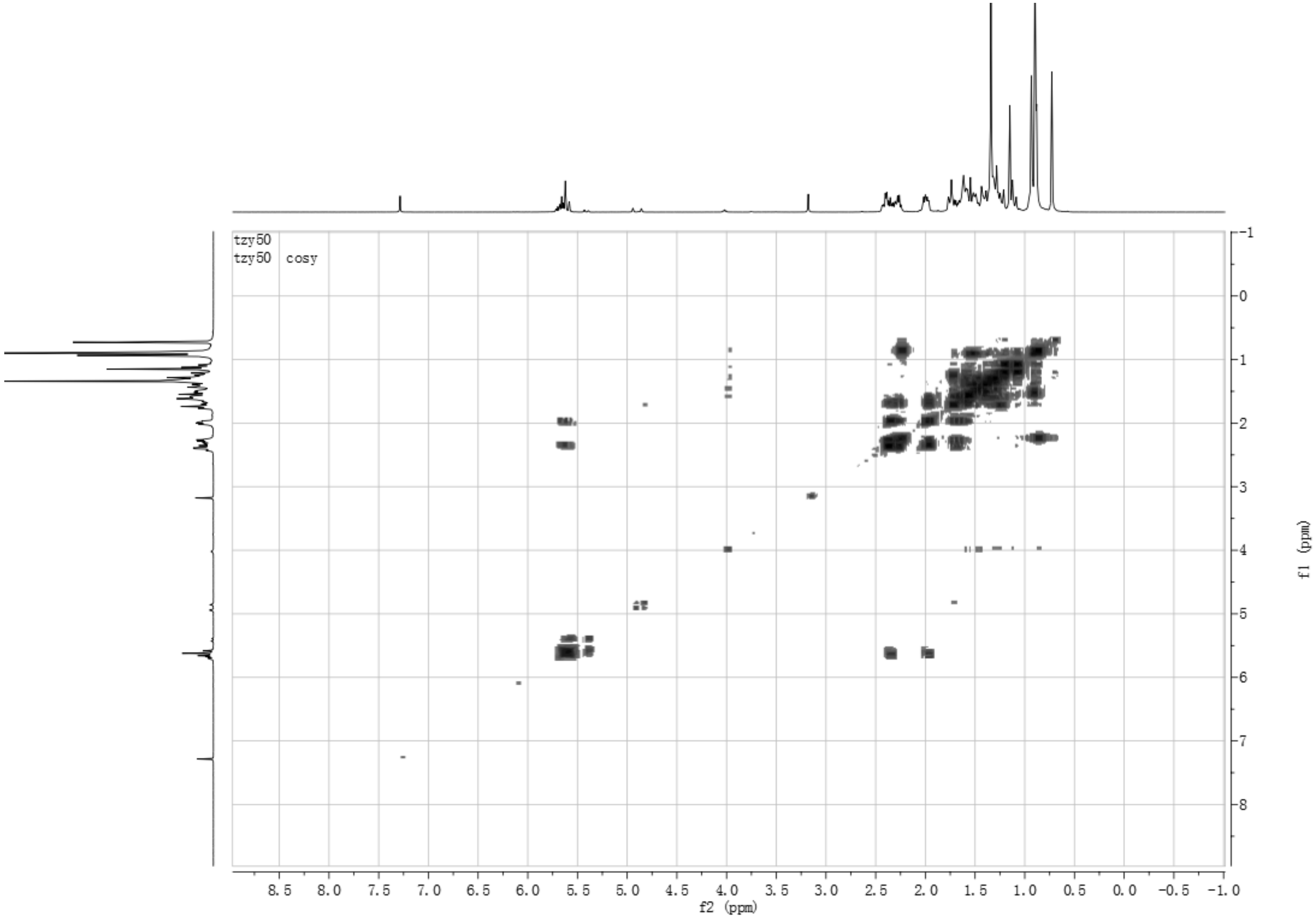

Figure S22. HMBC ($CDCl_3$, 500 MHz) spectrum of Astataricusone C (**3**).

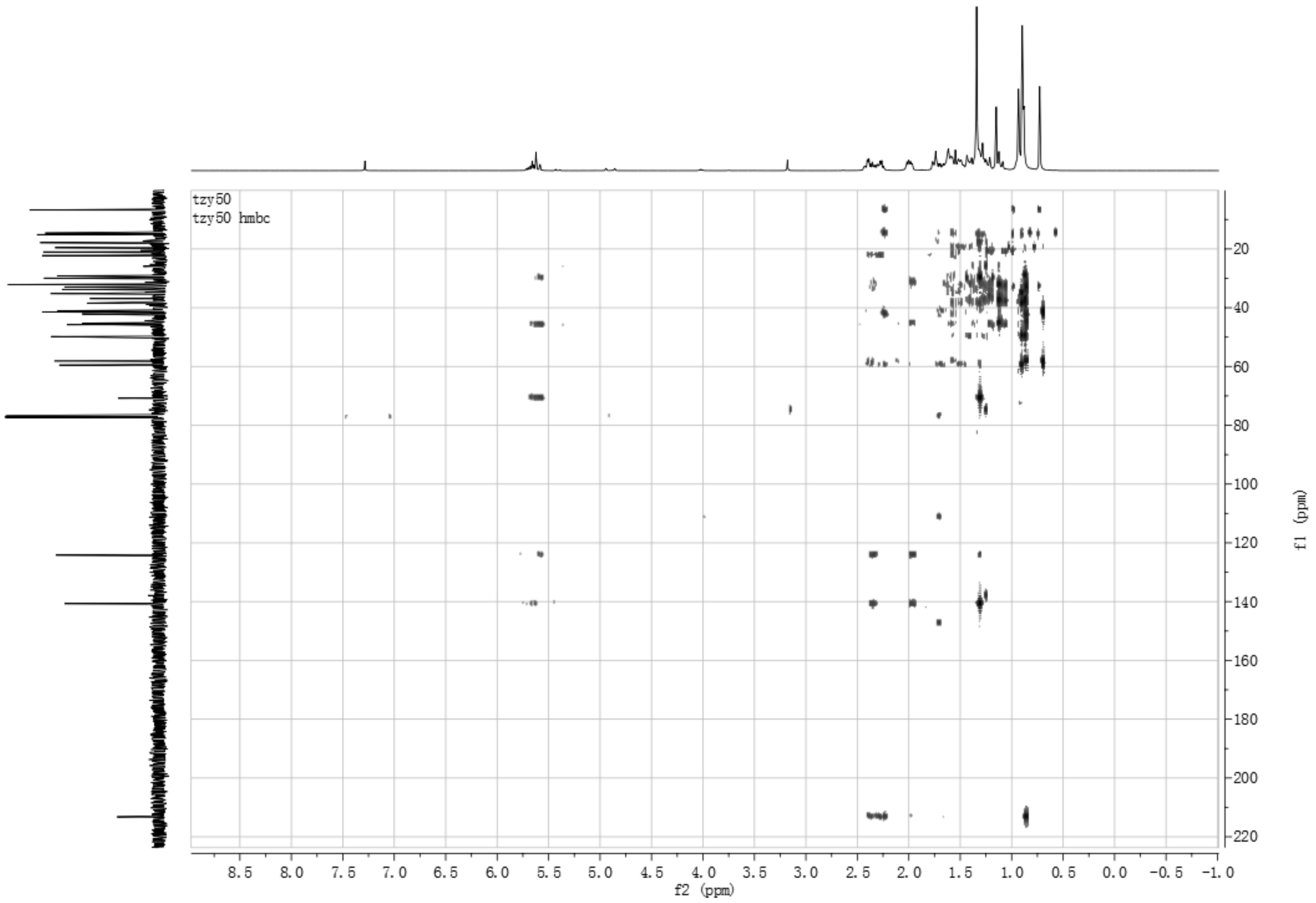

Figure S23. ROESY ($CDCl_3$, 500 MHz) spectrum of Astataricusone C (**3**).

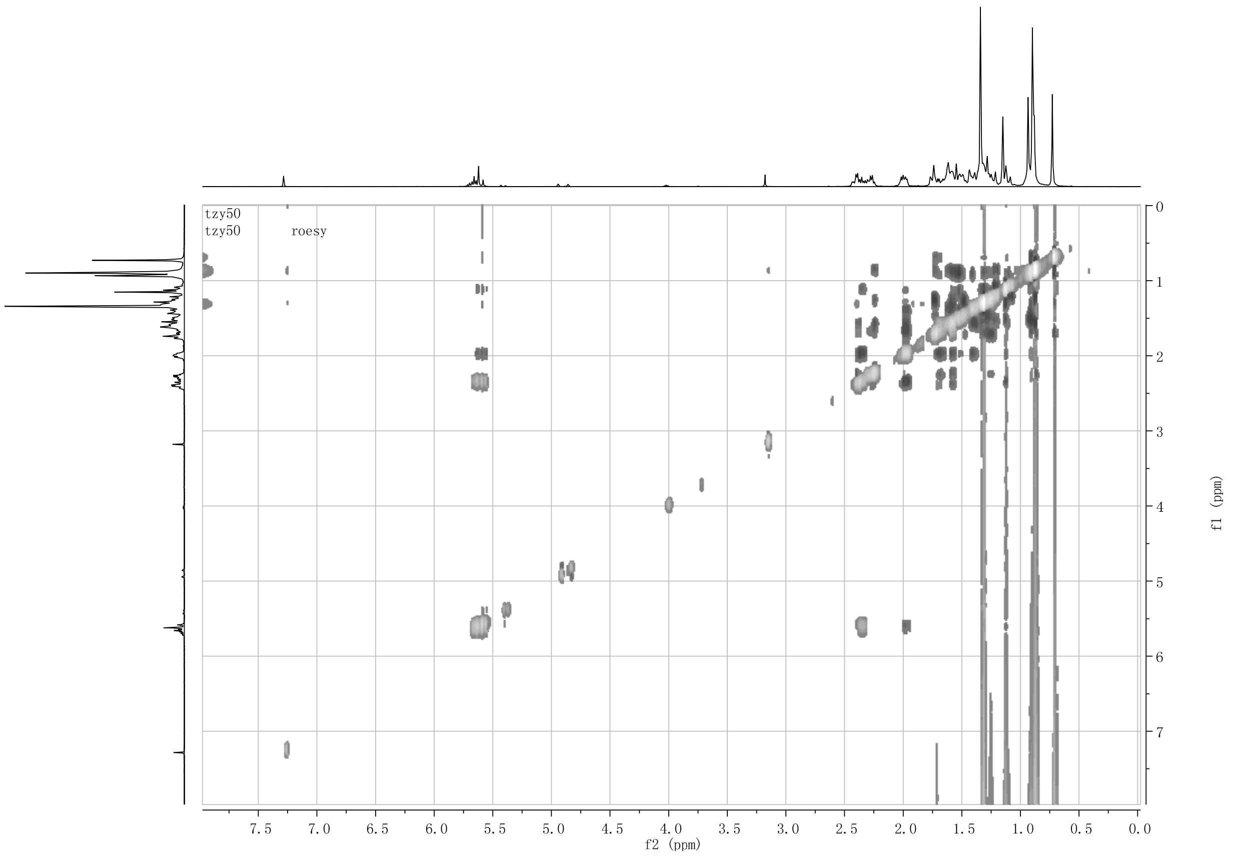

Figure S24. HREIMS spectrum of Astataricusone C (**3**).

Elemental Composition Report **Page 1**

Single Mass Analysis
Tolerance = 10.0 PPM / DBE: min = -10.0, max = 120.0
Selected filters: None

Monoisotopic Mass, Odd and Even Electron Ions
27 formula(e) evaluated with 1 results within limits (up to 51 closest results for each mass)
Elements Used:
C: 0-200 H: 0-400 O: 0-3

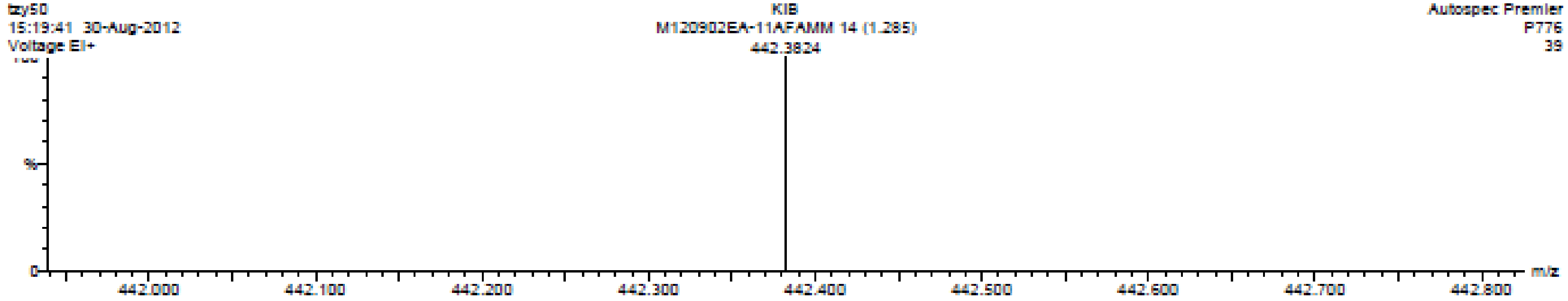

Minimum:				-10.0		
Maximum:		100.0	10.0	120.0		
Mass	Calc. Mass	mDa	PPM	DBE	i-FIT	Formula
442.3824	442.3811	1.3	2.9	6.0	5546039.0	C30 H50 O2

Figure S25. CD (MeOH) spectrum of Astataricusone C (**3**).

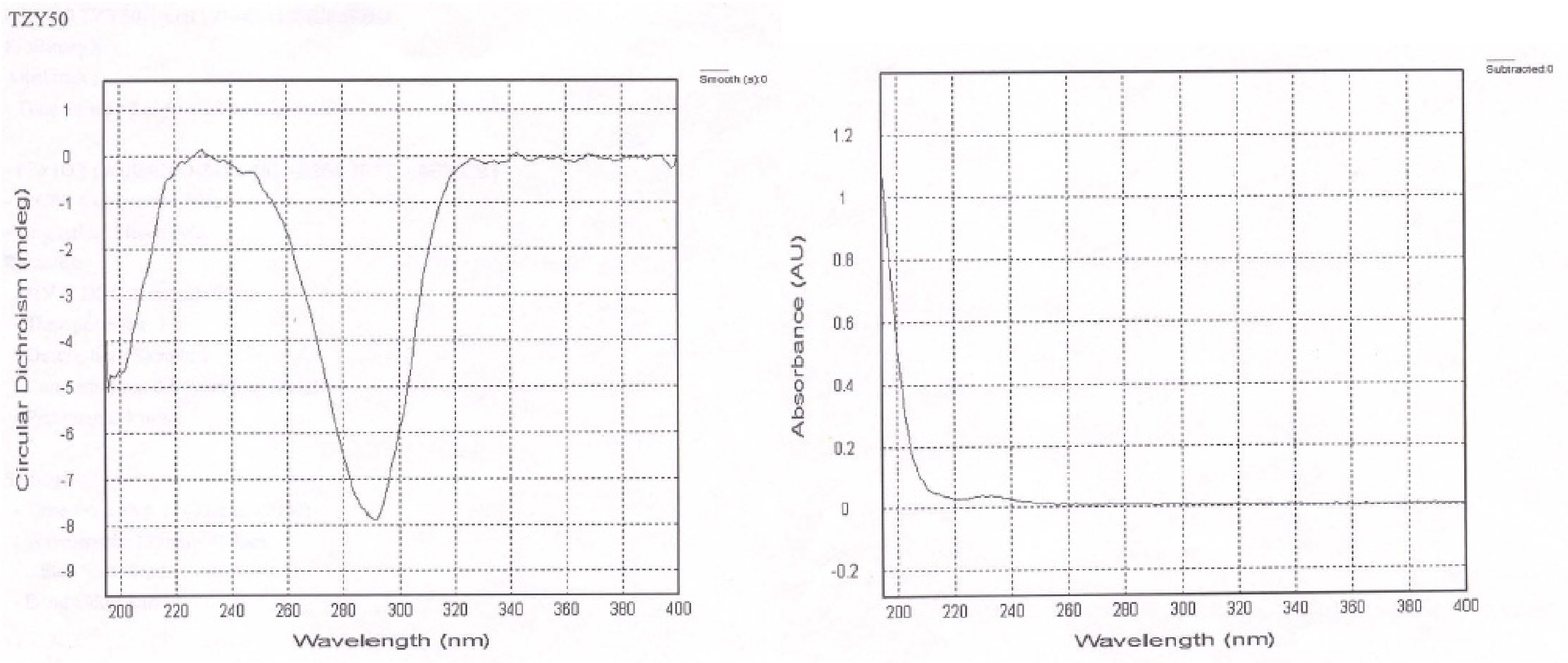

Figure S26. ^{1}H NMR ($CDCl_3$, 400 MHz) spectrum of Astataricusone D (**4**).

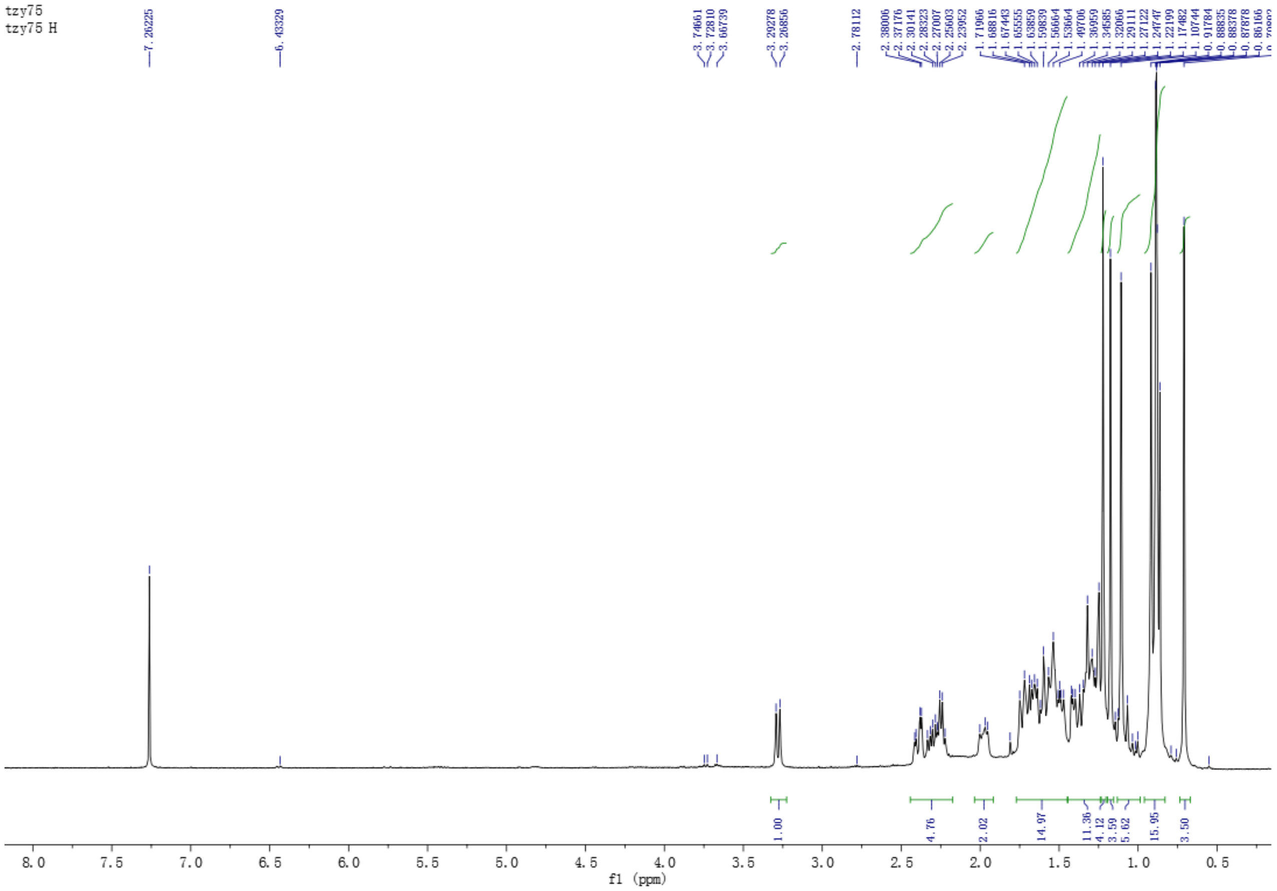

Figure S27. ^{13}C NMR ($CDCl_3$, 100 MHz) spectrum of Astataricusone D (**4**).

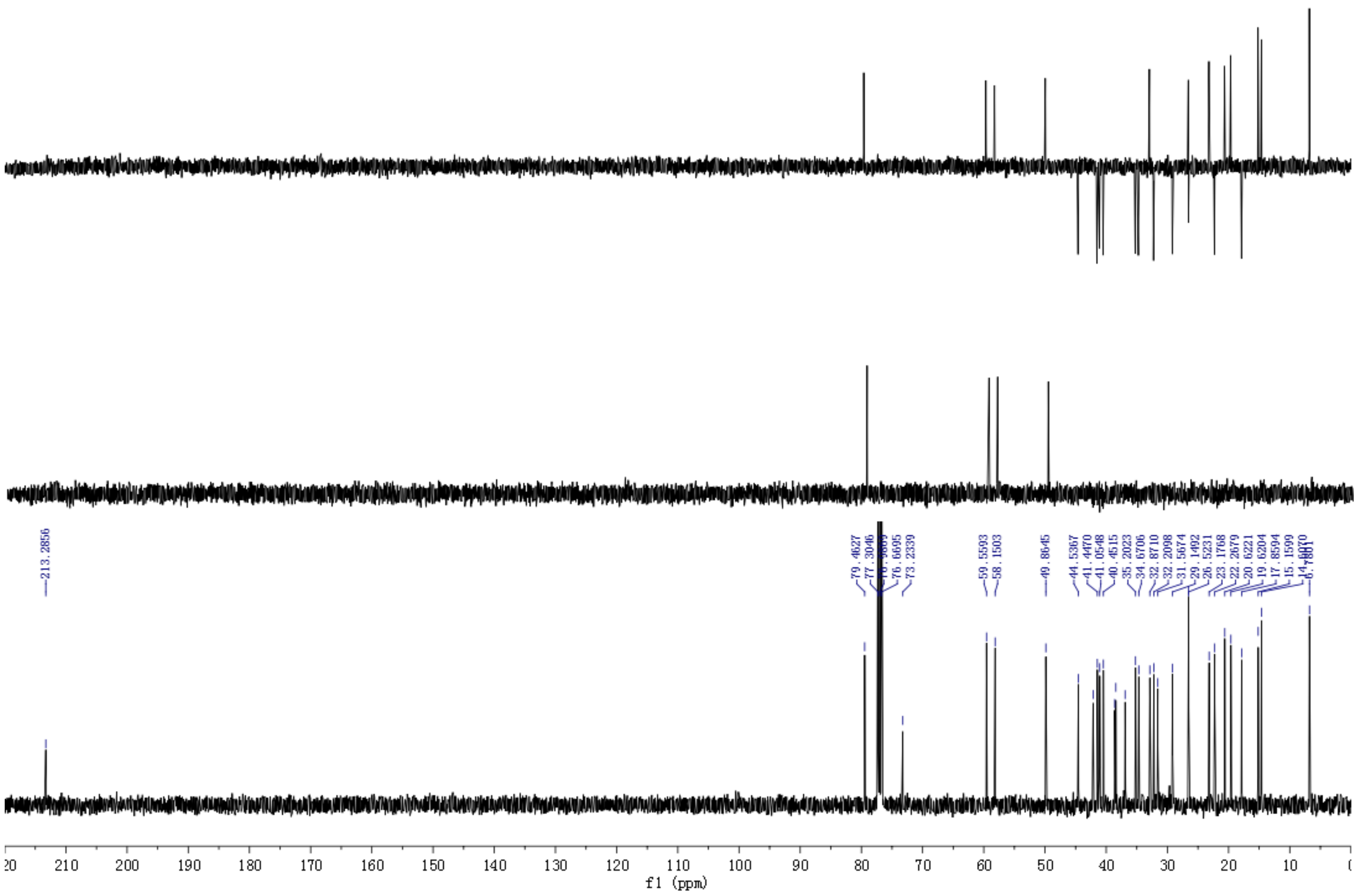

Figure S28. HSQC ($CDCl_3$, 500 MHz) spectrum of Astataricusone D (**4**).

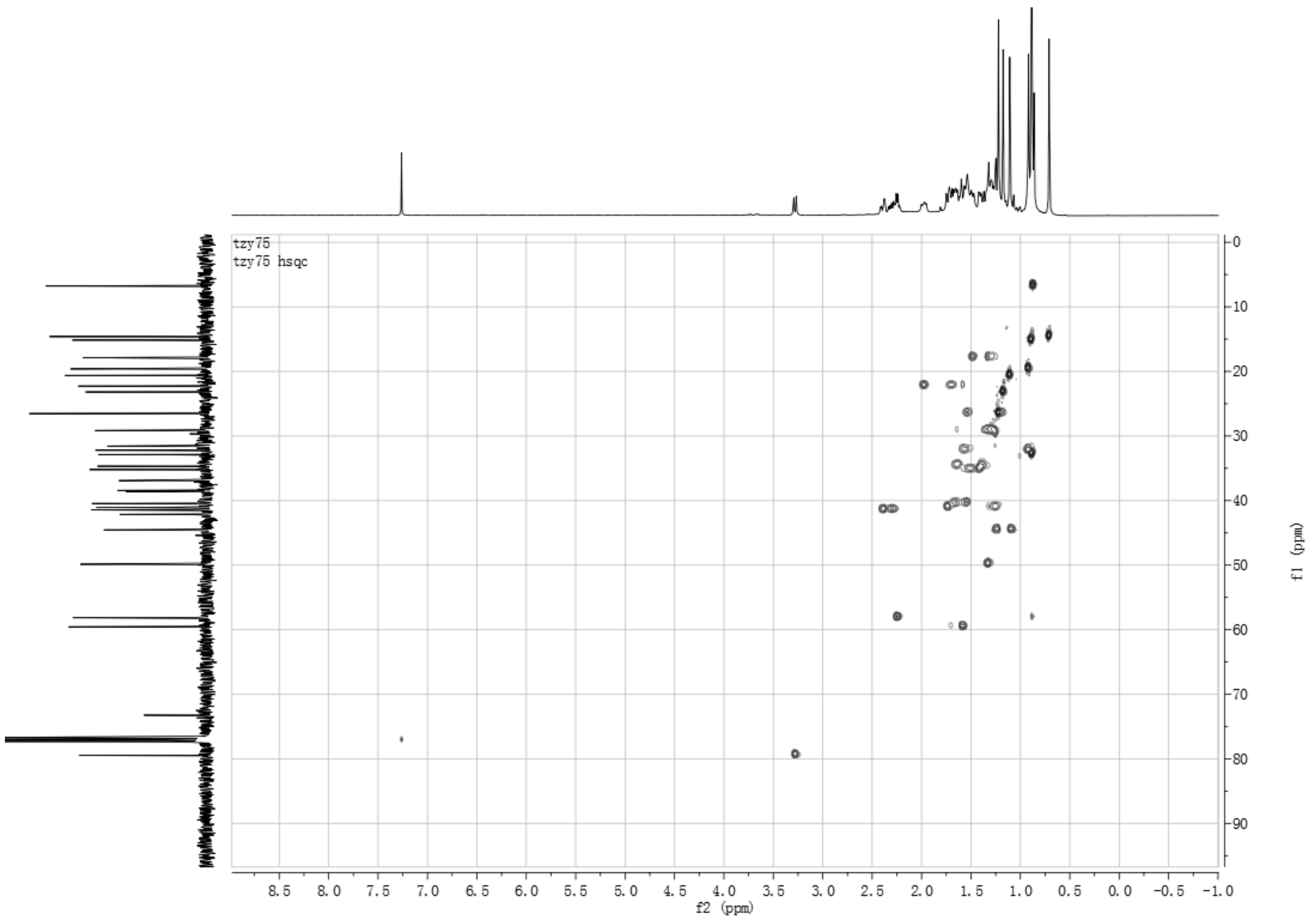

Figure S29. ^1H-^1H COSY ($CDCl_3$, 500 MHz) spectrum of Astataricusone D (**4**).

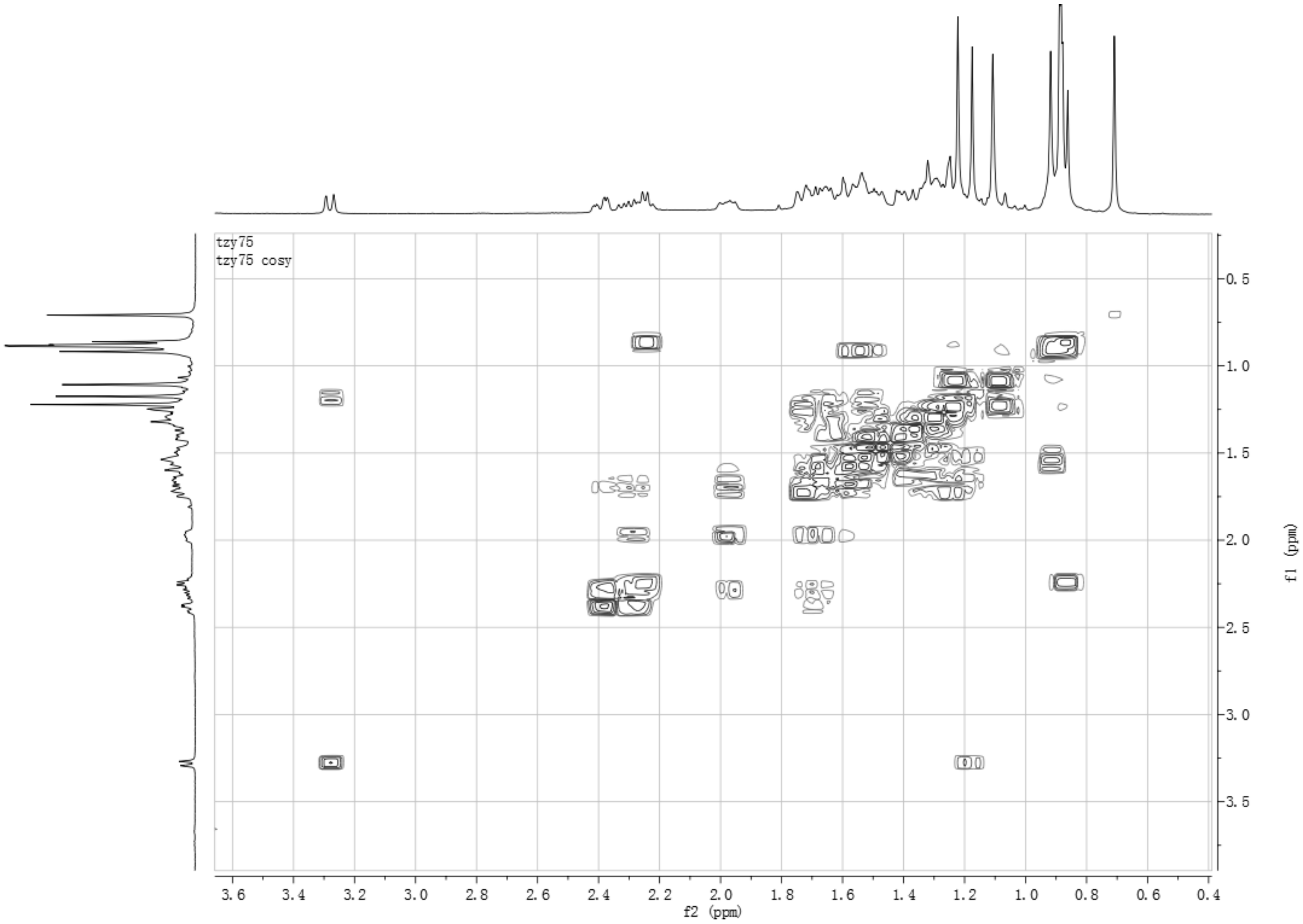

Figure S30. HMBC ($CDCl_3$, 500 MHz) spectrum of Astataricusone D (**4**).

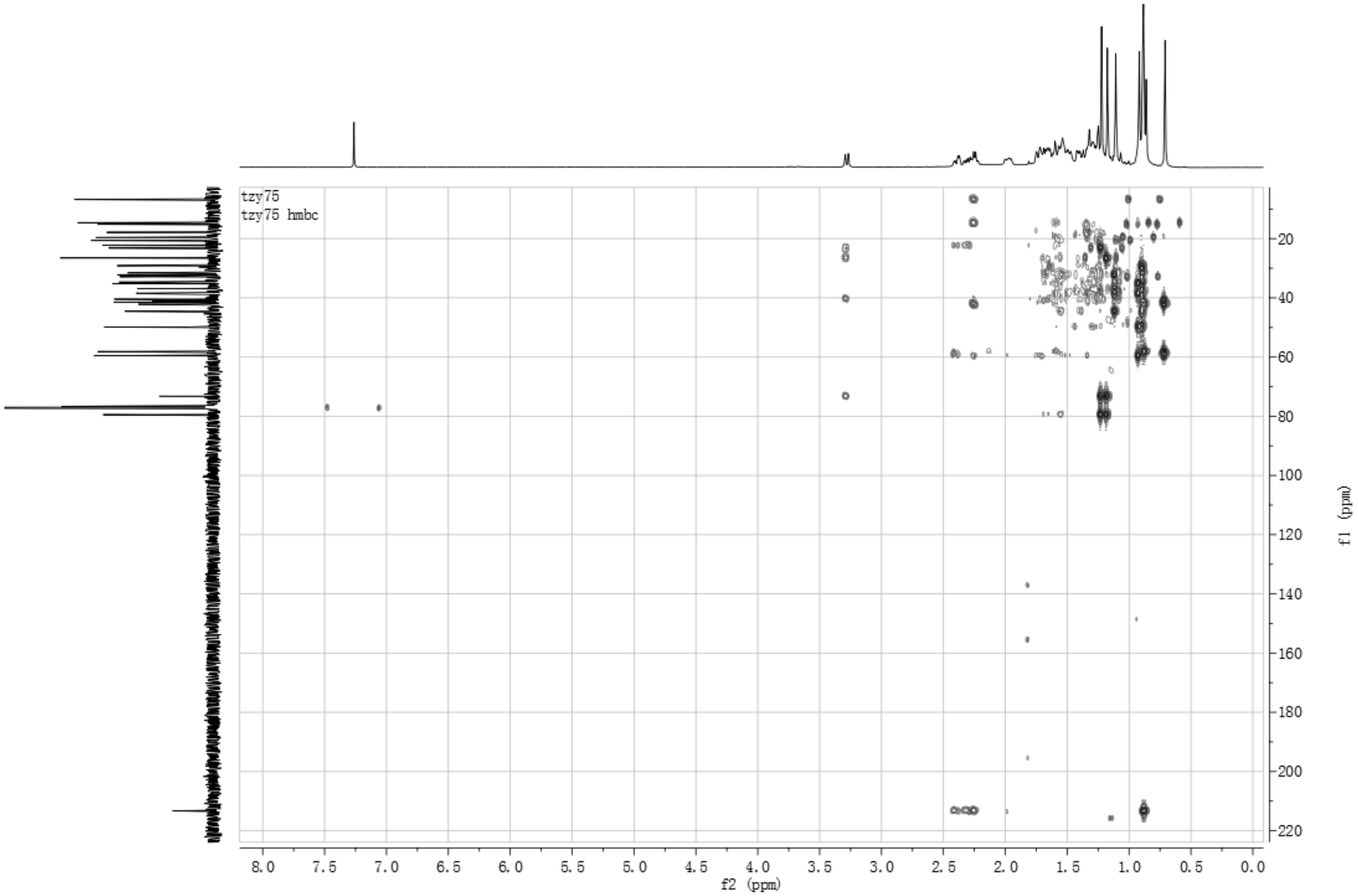

Figure S31. ROESY ($CDCl_3$, 500 MHz) spectrum of Astataricusone D (**4**).

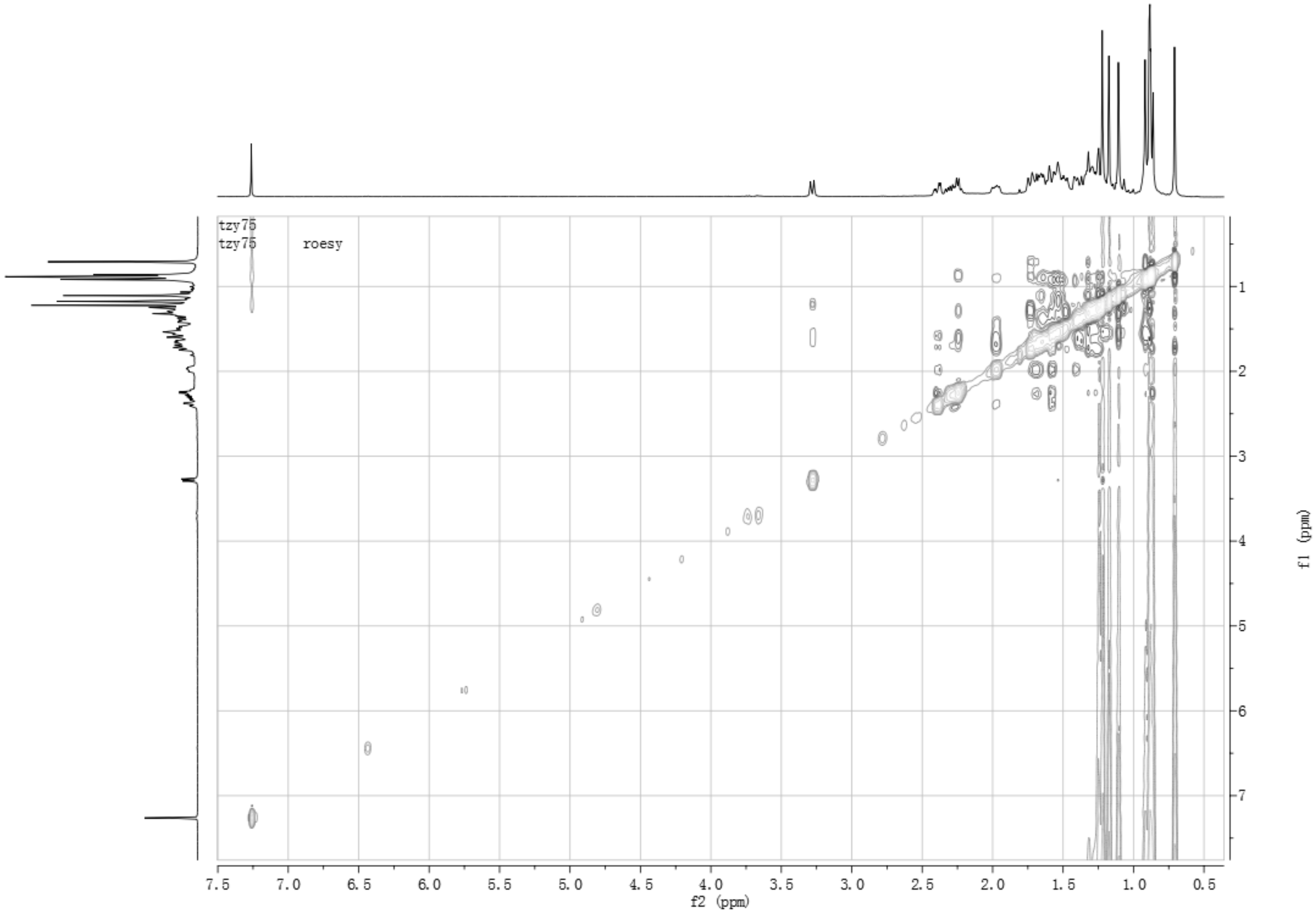

Figure S32. HREIMS spectrumof Astataricusone D (**4**).

Elemental Composition Report

Page 1

Single Mass Analysis

Tolerance = 10.0 PPM / DBE: min = -10.0, max = 120.0
Selected filters: None

Monoisotopic Mass, Odd and Even Electron Ions
30 formula(e) evaluated with 1 results within limits (up to 51 closest results for each mass)
Elements Used:
C: 0-200 H: 0-400 O: 0-3

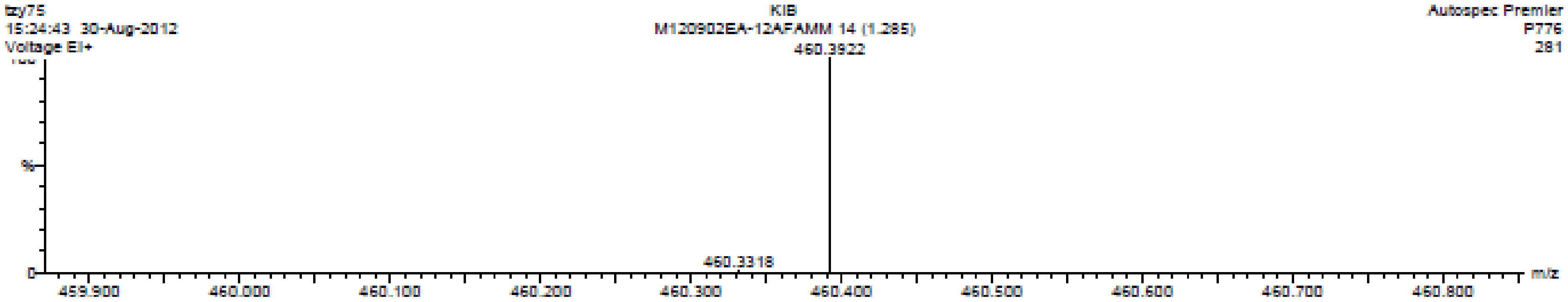

Minimum:				-10.0		
Maximum:		100.0	10.0	120.0		
Mass	Calc. Mass	mDa	PPM	DBE	i-FIT	Formula
460.3922	460.3916	0.6	1.3	5.0	5546158.5	C30 H52 O3

Figure S33. CD (DMSO) spectrum of Astataricusone D (**4**).

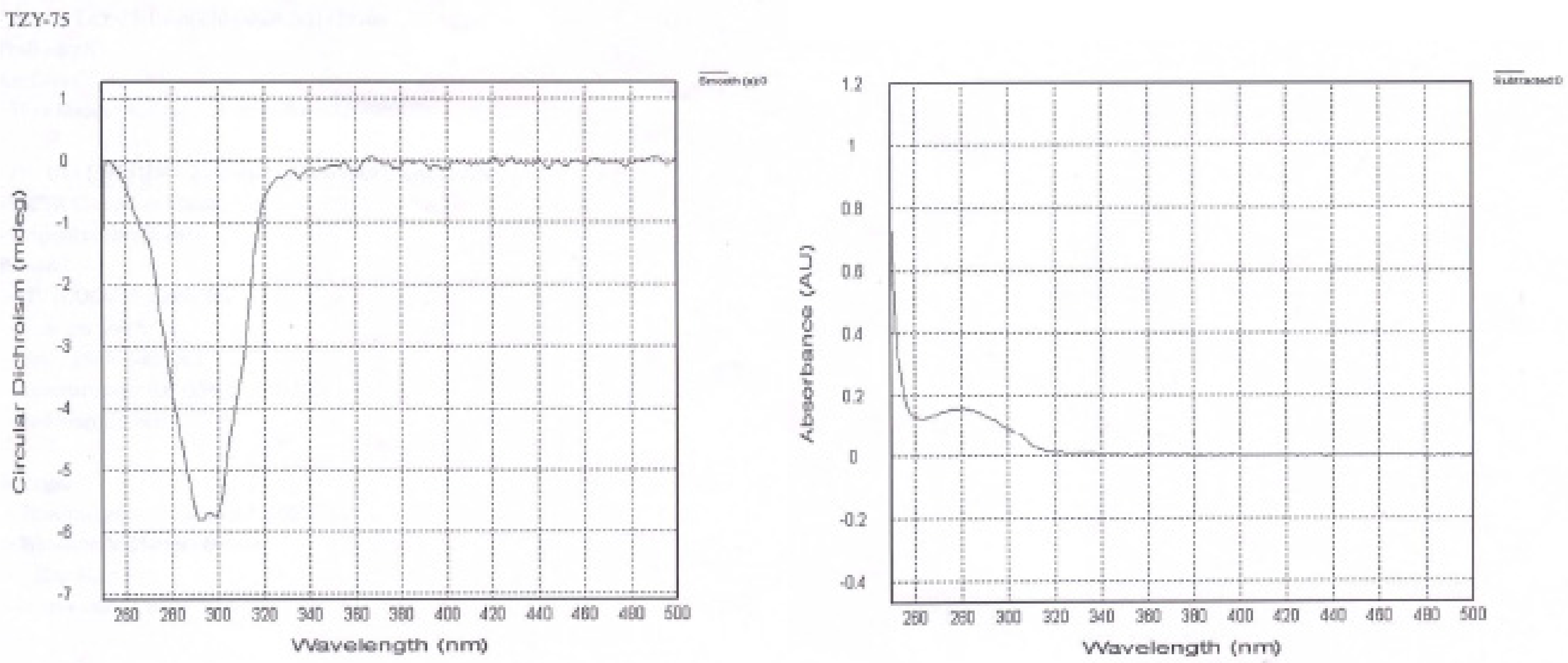

Figure S34. ICD (DMSO) spectrum of Astataricusone D (**4**).

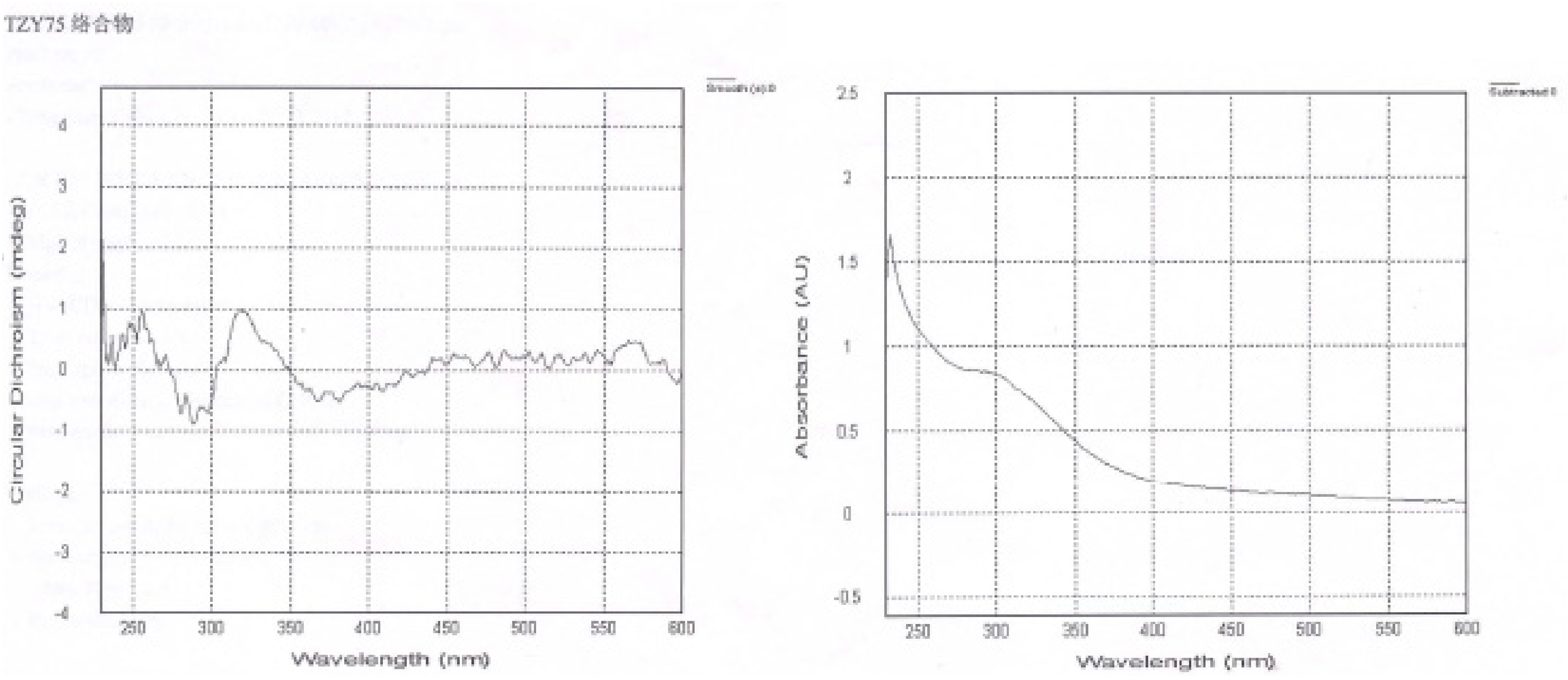

Figure S35. ^{1}H NMR ($CDCl_3$, 400 MHz) spectrum of Astataricusol A (**5**).

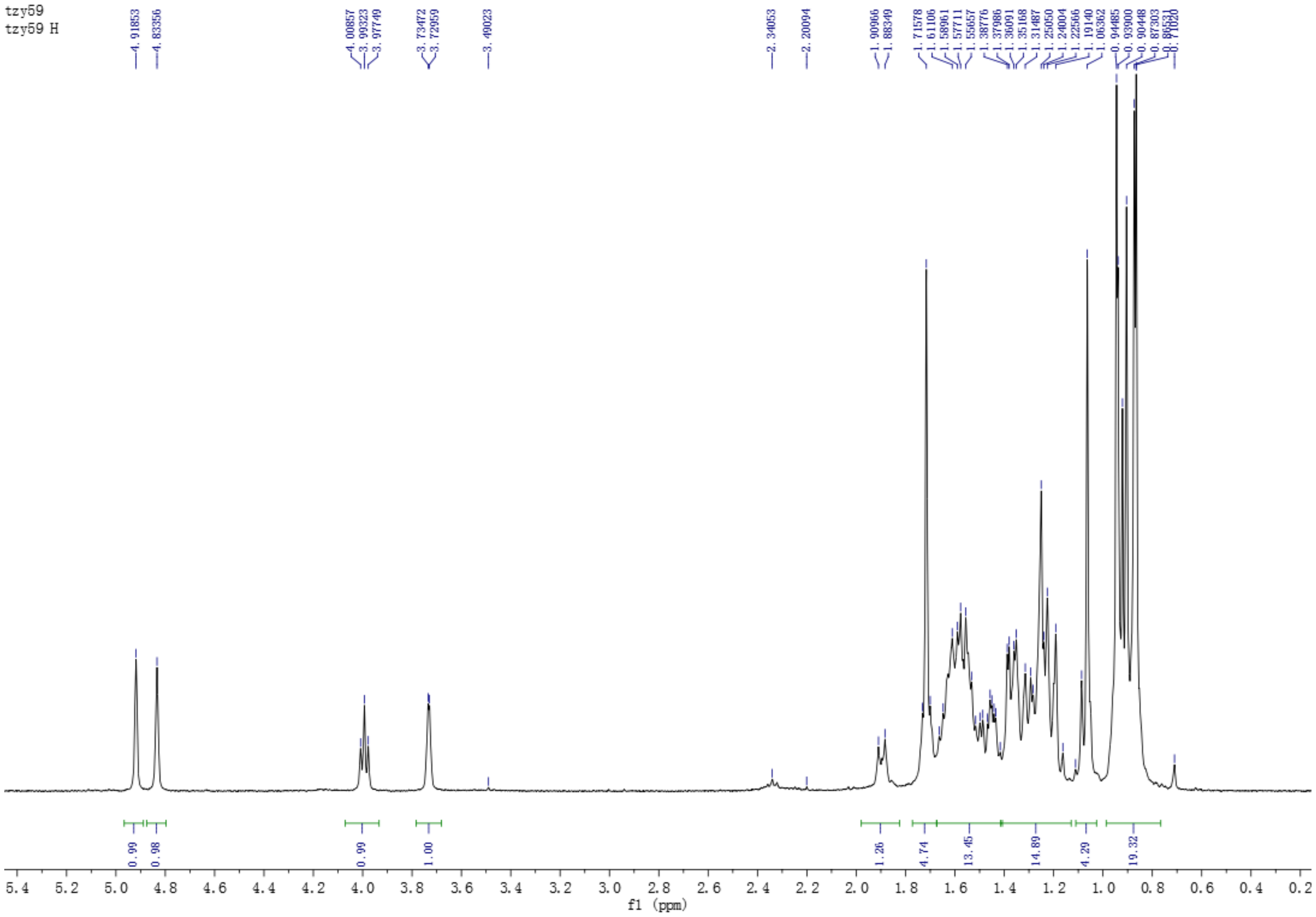

Figure S36. ^{13}C NMR ($CDCl_3$, 100 MHz) spectrum of Astataricusol A (**5**).

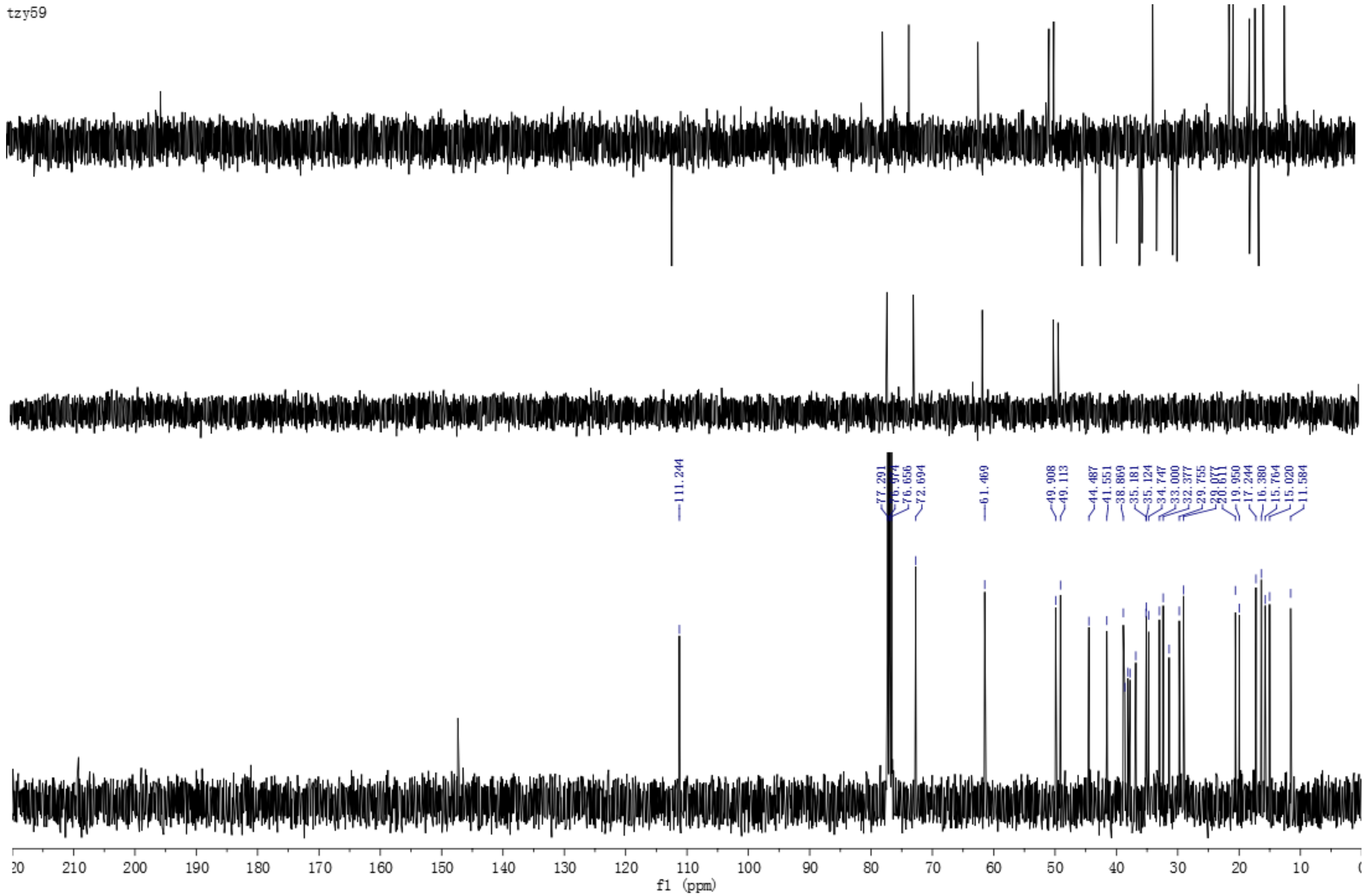

Figure S37. HSQC ($CDCl_3$, 500 MHz) spectrum of Astataricusol A (**5**).

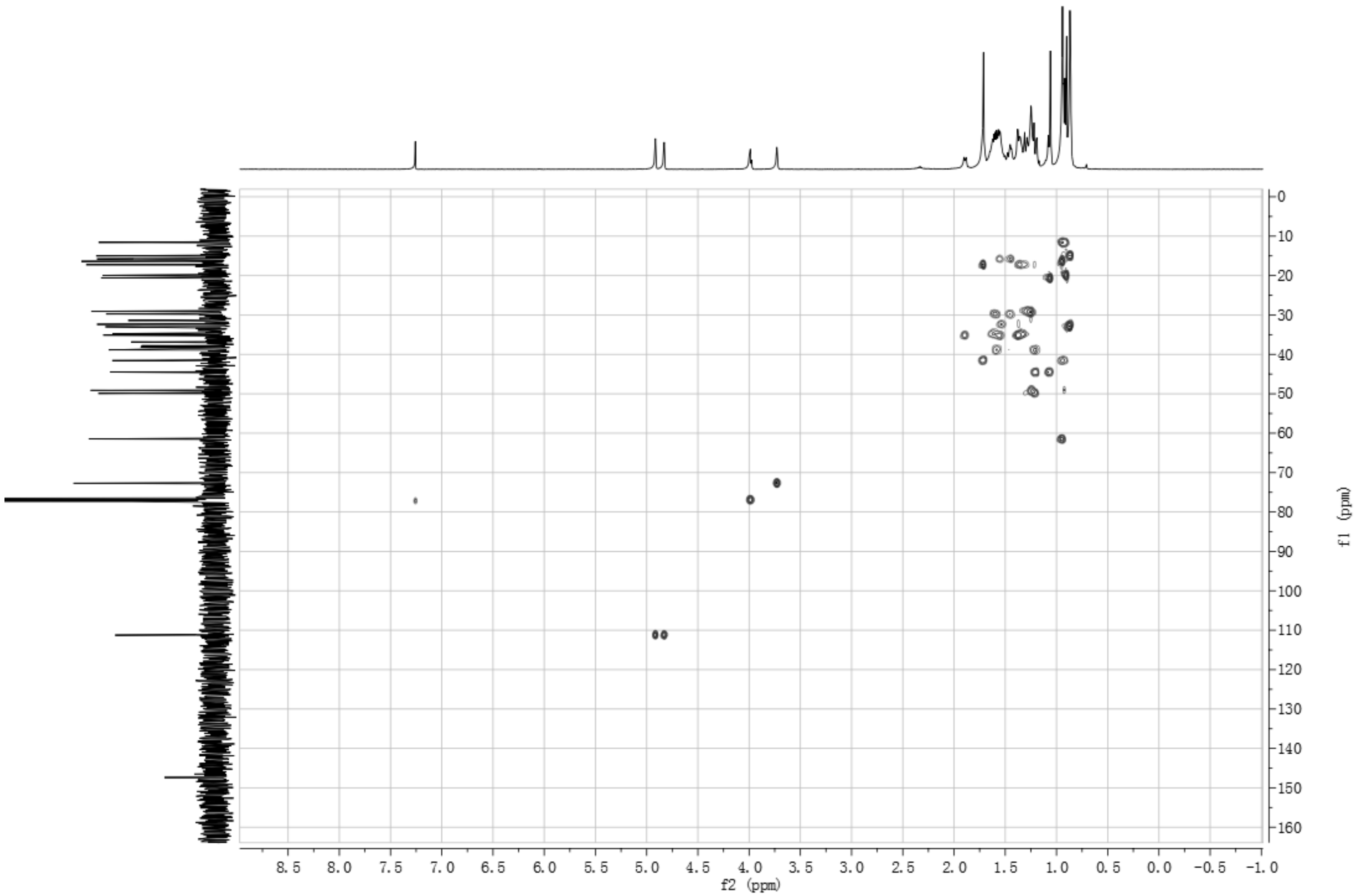

Figure S38. ^1H-^1H COSY ($CDCl_3$, 500 MHz) spectrum of Astataricusol A (**5**).

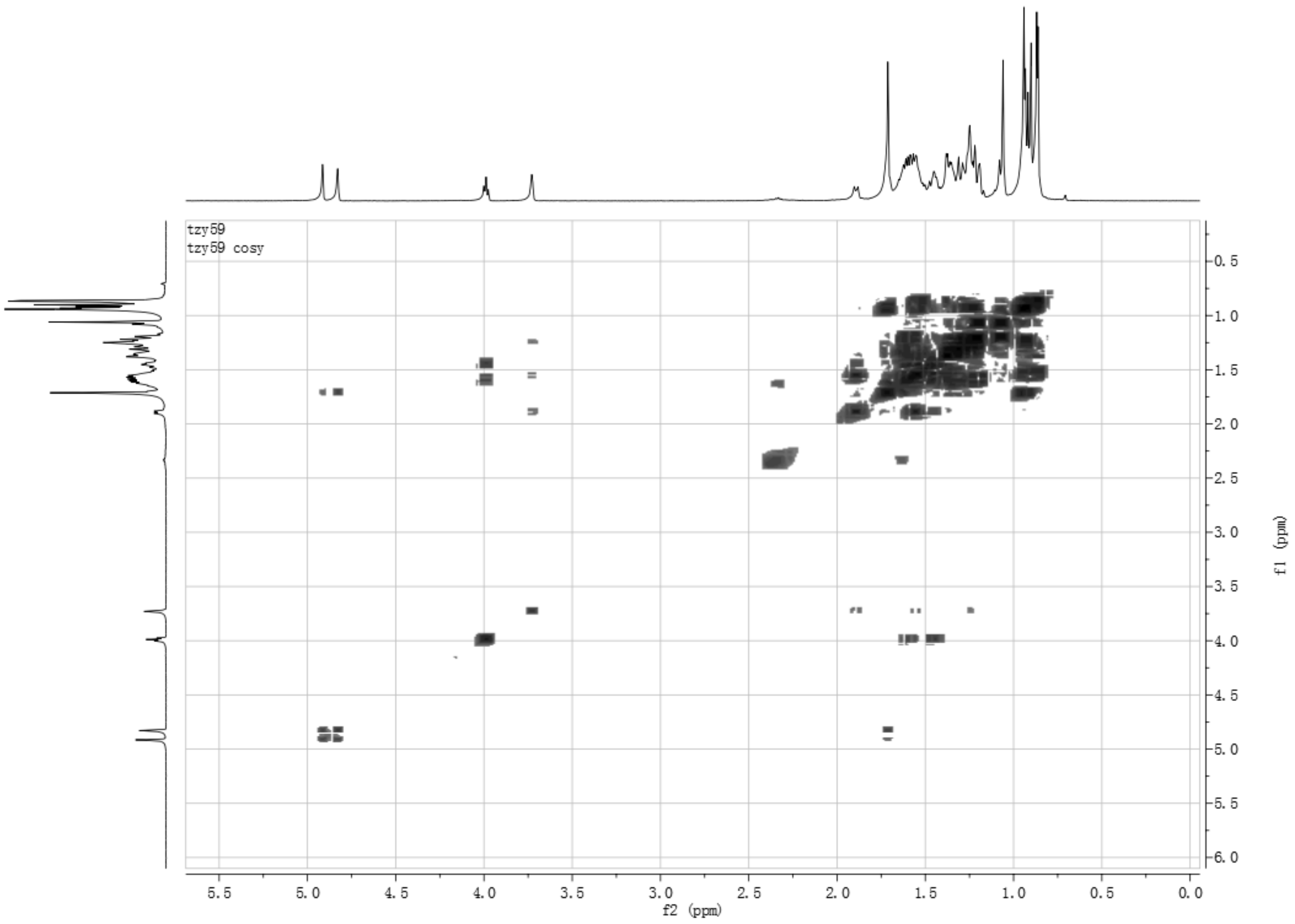

Figure S39. HMBC ($CDCl_3$, 500 MHz) spectrum of Astataricusol A (**5**).

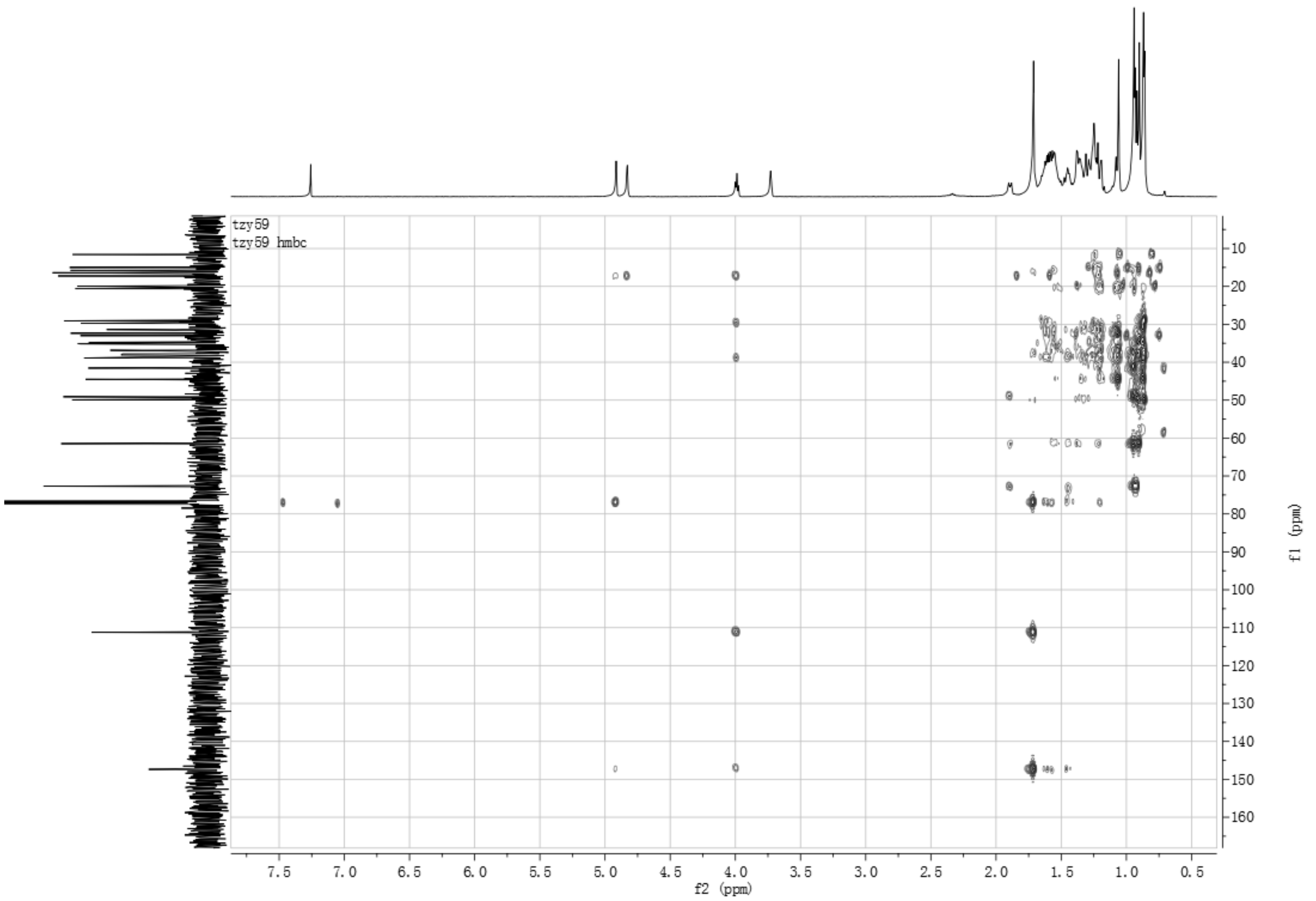

Figure S40. ROESY ($CDCl_3$, 500 MHz) spectrum of Astataricusol A (**5**).

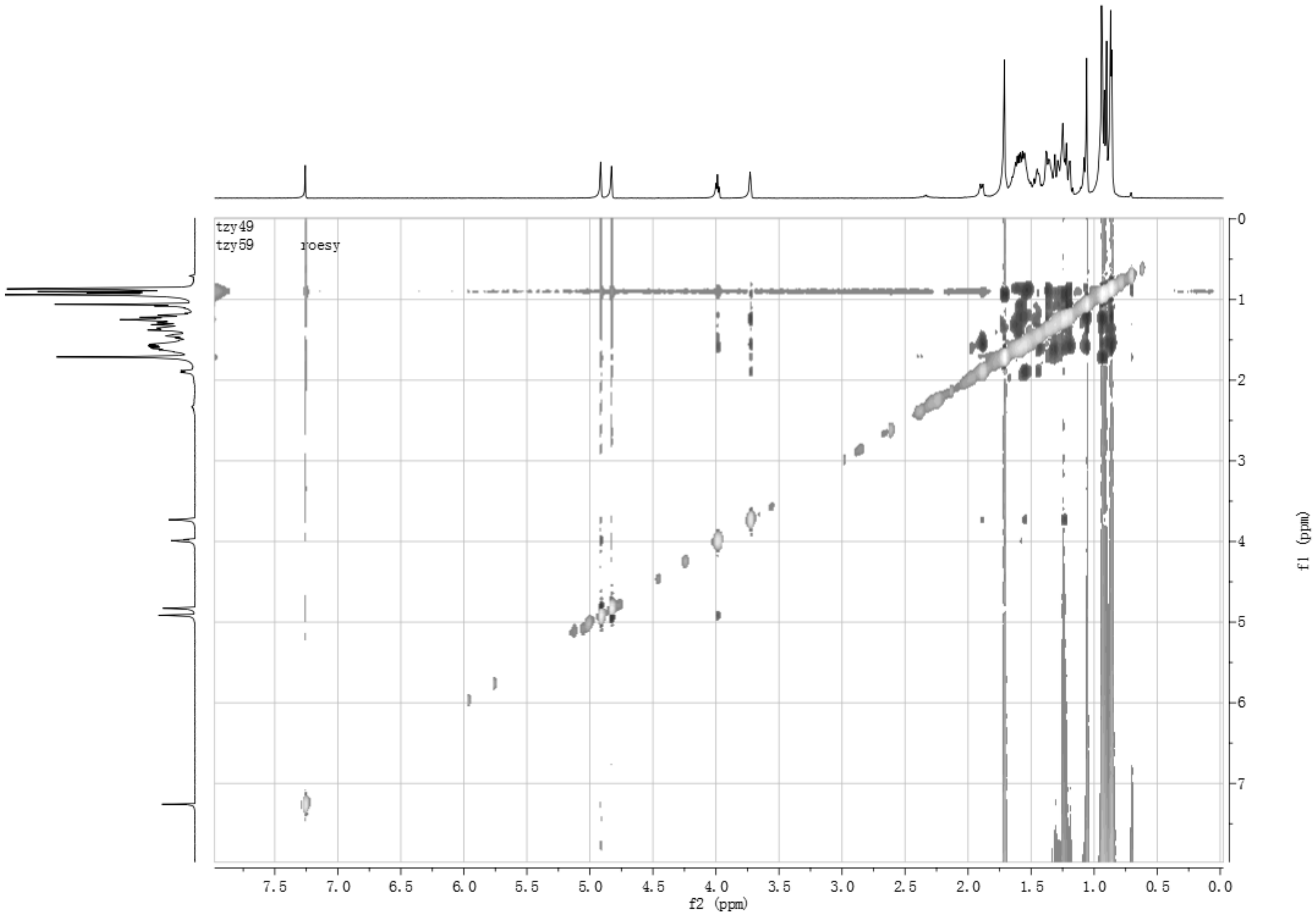

Figure S41. HREIMS spectrum of Astataricusol A (**5**).

Elemental Composition Report **Page 1**

Single Mass Analysis
Tolerance = 10.0 PPM / DBE: min = -10.0, max = 120.0
Selected filters: None

Monoisotopic Mass, Odd and Even Electron Ions
29 formula(e) evaluated with 1 results within limits (up to 51 closest results for each mass)
Elements Used:
C: 0-200 H: 0-400 O: 0-3

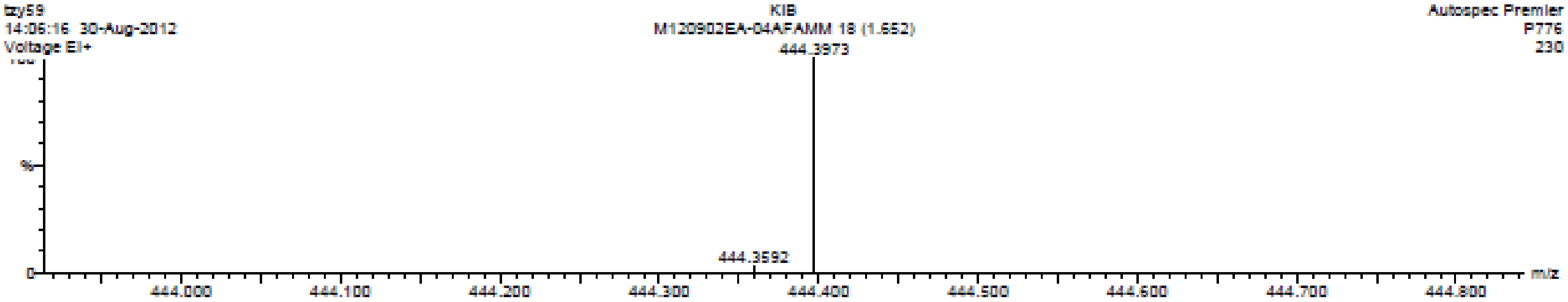

Minimum:				-10.0		
Maximum:		100.0	10.0	120.0		
Mass	Calc. Mass	mDa	PPM	DBE	i-FIT	Formula
444.3973	444.3967	0.6	1.4	5.0	5546133.5	C30 H52 O2

Figure S42. CD (MeOH) spectrum of Astataricusol A (**5**).

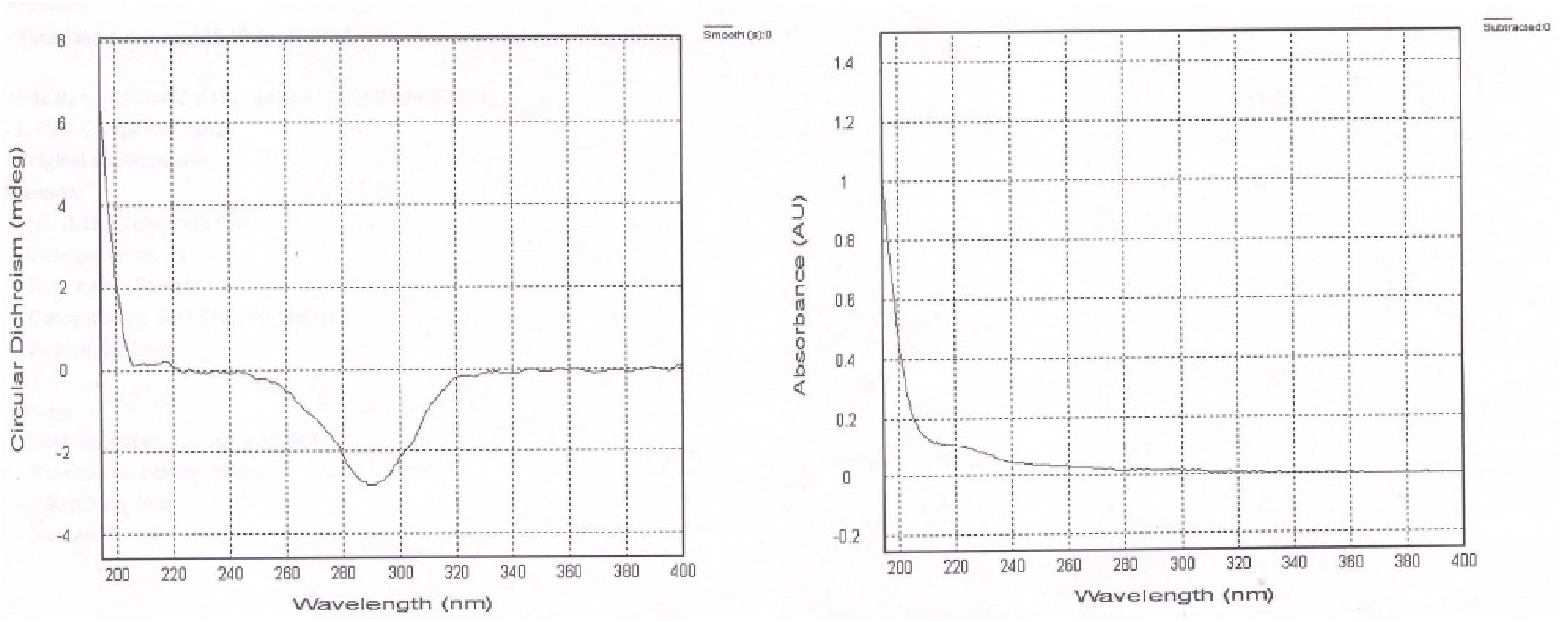

Figure S43. CD (MeOH) spectrum of Epishionol (**6**).

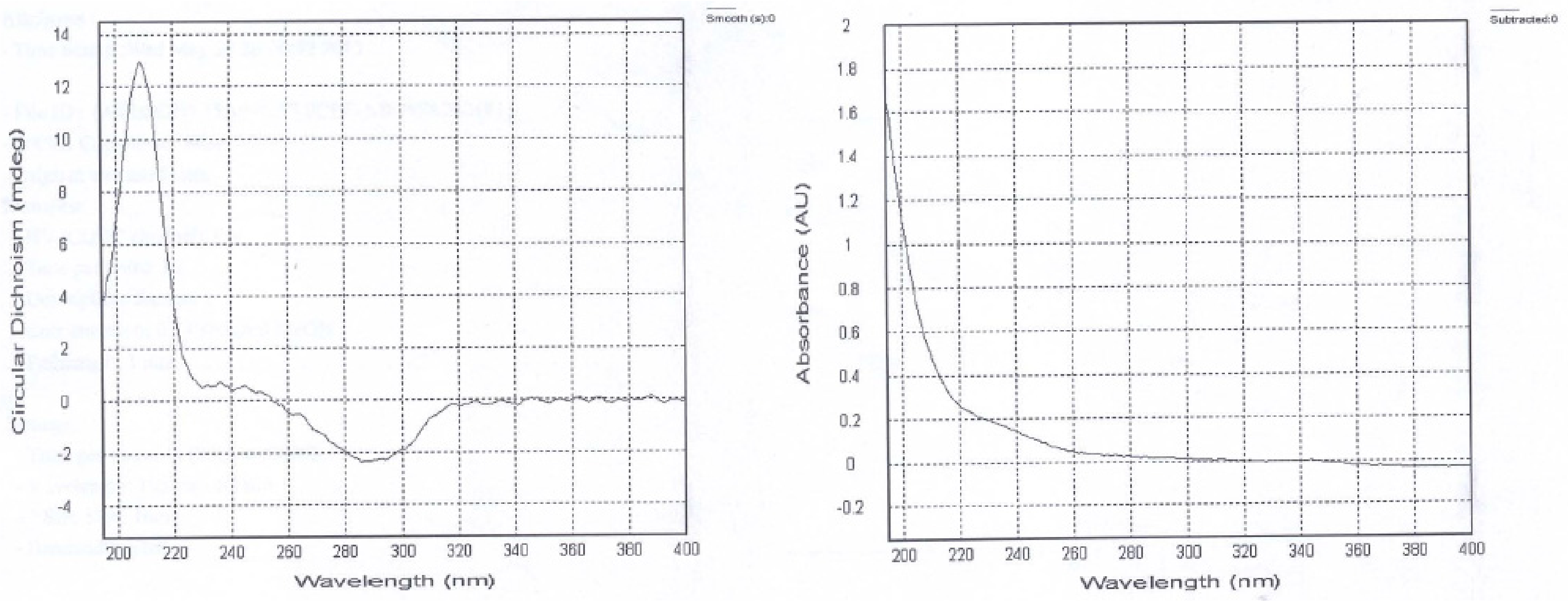

Supplement: Supplementary file 1 [file molecules-18-14585-s003.pdf]
